# Supplementary material for: GPCR kinase knockout cells reveal the impact of individual GRKs on arrestin binding and GPCR regulation
Source: Nat Commun. 2022 Jan 27;13:540. doi: 10.1038/s41467-022-28152-8 (PMC8795447; doi:10.1038/s41467-022-28152-8)
Supplement: Supplementary file 1 — Supplementary Information [file 41467_2022_28152_MOESM1_ESM.pdf]

GPCR kinase knockout cells reveal the impact of individual GRKs on arrestin binding and GPCR regulation

J. Drube*, R.S. Haider*, E.S.F. Matthees, M. Reichel, J. Zeiner, S. Fritzwanker, C. Ziegler, S. Barz, L. Klement, J. Filor, V. Weitzel, A. Kliewer, E. Miess-Tanneberg, E. Kostenis, S. Schulz, and C. Hoffmann

* contributed equally
to whom correspondence should be addressed: carsten.hoffmann@med.uni-jena.de

Supplementary Information

This .pdf includes:
Supplementary Figures 1-15
Supplementary Tables 1-5
Supplementary References

Supplementary Figure 1

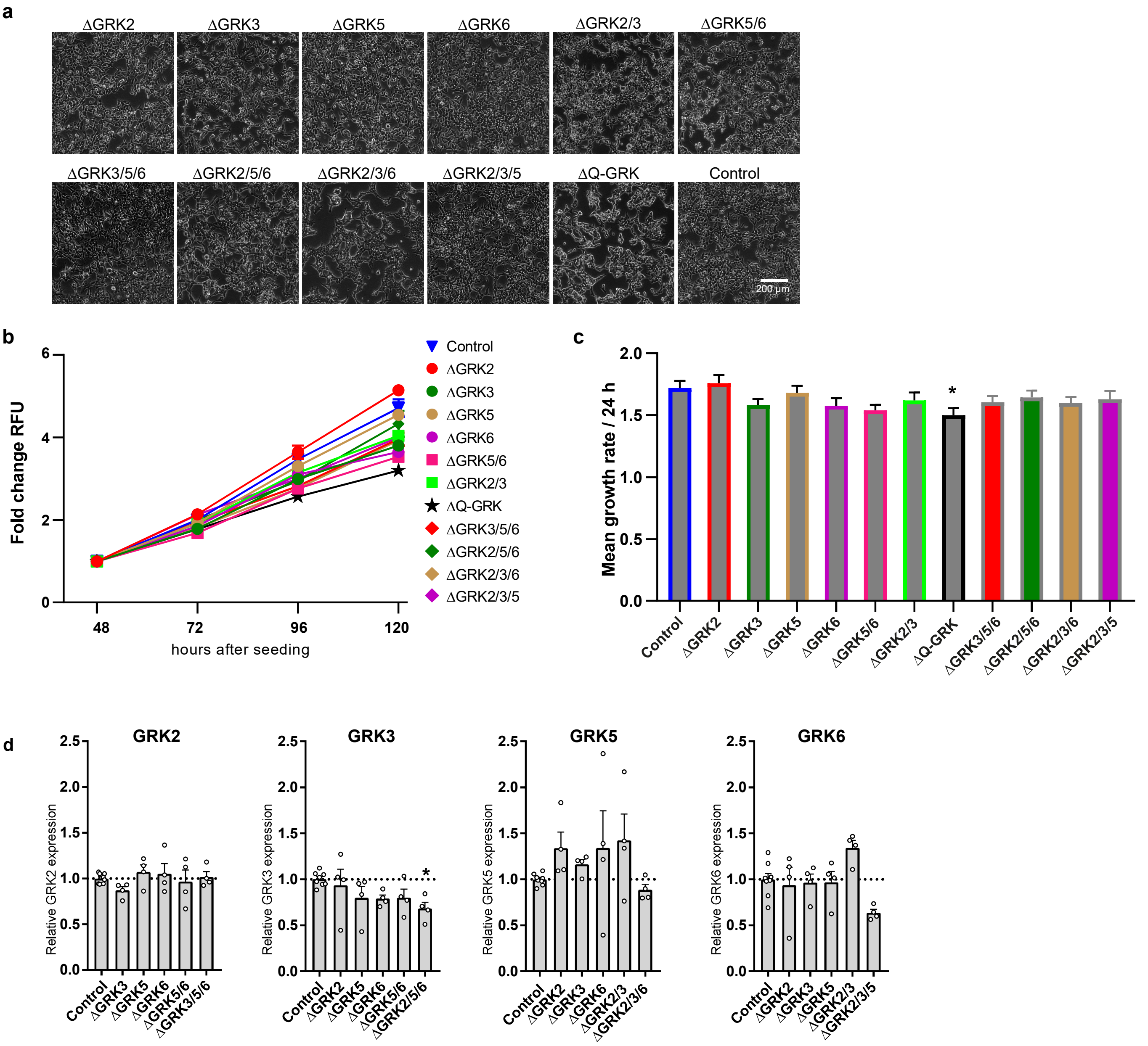

Supplementary Figure 1: Characterisation of GRK knockout cell properties. a Single (ΔGRK2, 3, 5, 6), double (ΔGRK2/3; 5/6), triple (ΔGRK3/5/6, 2/5/6, 2/3/6, 2/3/5), and quadruple (ΔQ-GRK) GRK knockout cells were generated using the CRISPR/Cas9 technology and established as single cell clones. Cell morphology was examined regularly. Representative images acquired by phase contrast microscopy are shown. **b** Cell viability was determined by adding CellTiter-Blue reagent (Promega) at indicated timepoints and measuring relative fluorescence units (RFU) after 90 minutes of incubation. Shown are the respective growth curves as mean of n = 3 independent experiments ± SEM, expressed as fold change compared to 48 h after seeding. **c** Growth rates per 24 h were calculated from data shown in **b** for each cell clone and are presented in bar graphs as mean ± SEM. Growth rates of generated cell clones were compared to the growth rate of Control cells using ANOVA and two-sided Dunnett's test (* $p < 0.05$). **d** Quantification of the untargeted GRKs was performed by analysing four independent Western blots (representative blot in Figure 1a) made from n = 4 independent protein lysates of each cell clone. Data are depicted as mean ± SEM, normalised to the respective remaining expression in Control cells. GRK expression levels were compared to expression in Control cells using ANOVA and two-sided Dunnett's test (* $p < 0.05$). All exact p values, test statistics, effect sizes, confidence intervals, and degrees of freedom are provided in the Source Data files.

Supplementary Figure 2

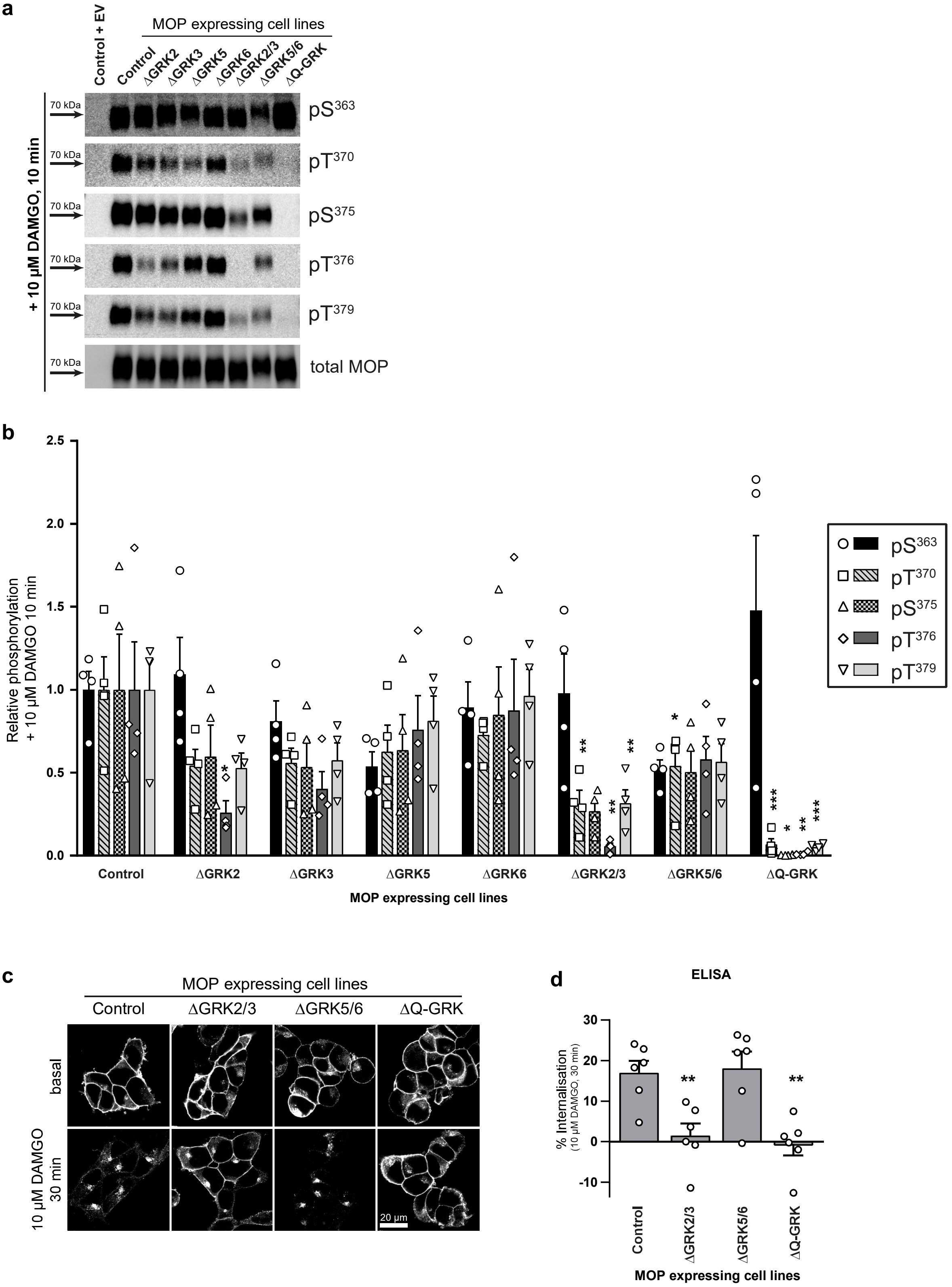

Supplementary Figure 2: GRK knockout cells confirm GRK-specific µ-opioid receptor (MOP) phosphorylation. a-d ΔGRK clones and Control cells stably expressing an N-terminally HA-tagged MOP were used for analysis of site-specific phosphorylation and receptor internalisation. **a** Prior to immunoprecipitation, cells were treated with the MOP agonist [D-Ala2, N-MePhe4; Gly-ol]-enkephalin (DAMGO) as indicated and subsequently analysed for site-specific phosphorylation by Western blot. Shown are representative blots of $n = 4$ independent experiments. **b** Blots were quantified and relative phosphorylation compared to the respective Control signal of the indicated GRK target sites is shown as mean ± SEM. Statistical significance compared to signal obtained in Control cells was determined by ANOVA and two-sided Dunnett's test (* $p < 0.05$; ** $p < 0.01$; *** $p < 0.001$). **c** ΔGRK MOP cells were stimulated with DAMGO as indicated or left untreated (basal). After fixation, the cells were stained with anti-HA antibody followed by Alexa488-conjugated secondary antibody, and examined by confocal microscopy. Shown are representative images of $n = 5$ independent experiments. **d** Receptor internalisation was measured by ELISA. Data represent per cent loss of cell-surface receptors in DAMGO treated cells as compared to vehicle stimulated sister cultures. Data are presented as mean of $n = 6$ independent experiments ± SEM. Differences in internalisation between indicated cell clones and Control cells were statistically analysed using ANOVA and two-sided Dunnett's test (** $p < 0.01$). All exact p values, test statistics, effect sizes, confidence intervals, and degrees of freedom are provided in the Source Data files.

Supplementary Figure 3

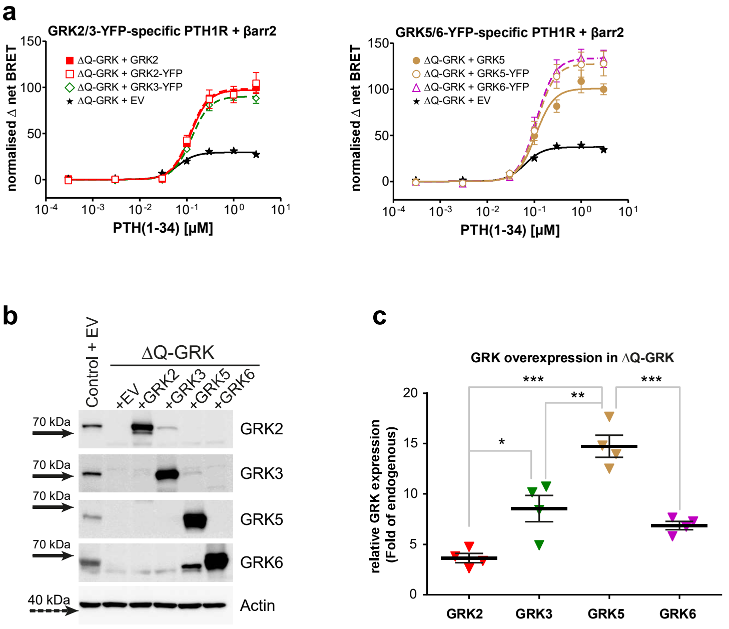

Supplementary Figure 3: Verification of GRK-YFP-mediated β-arrestin recruitment and quantification of GRK overexpression levels. a ΔQ-GRK cells were transfected with either YFP-tagged or untagged GRKs as indicated and β-arrestin2 (βarr2) recruitment to the parathyroid hormone 1 receptor (PTH1R) was measured using our NanoBRET βarr recruitment assay. Data points are presented as the mean ± SEM of the calculated Δ net BRET fold change from n = 3 independent experiments. The utilisation of the GRK-YFP constructs resulted in comparable βarr2 recruitment as in presence of the untagged GRKs (corresponds to Figure 1f). **b, c** Representative Western blot (**b**) and quantification (**c**) of n = 4 independent experiments showing the overexpression of GRK 2, 3, 5, or 6 in ΔQ-GRK in comparison to endogenous levels (Control + empty vector (EV)). The actin-normalised values are represented as fold expression of respective signal from endogenously expressed GRKs detected on the same membrane. GRK fold overexpressions were compared using ANOVA and two-sided Tukey's test (* $p < 0.05$; ** $p < 0.01$; *** $p < 0.001$). All exact p values, test statistics, effect sizes, confidence intervals, and degrees of freedom are provided in the Source Data files

Supplementary Figure 4

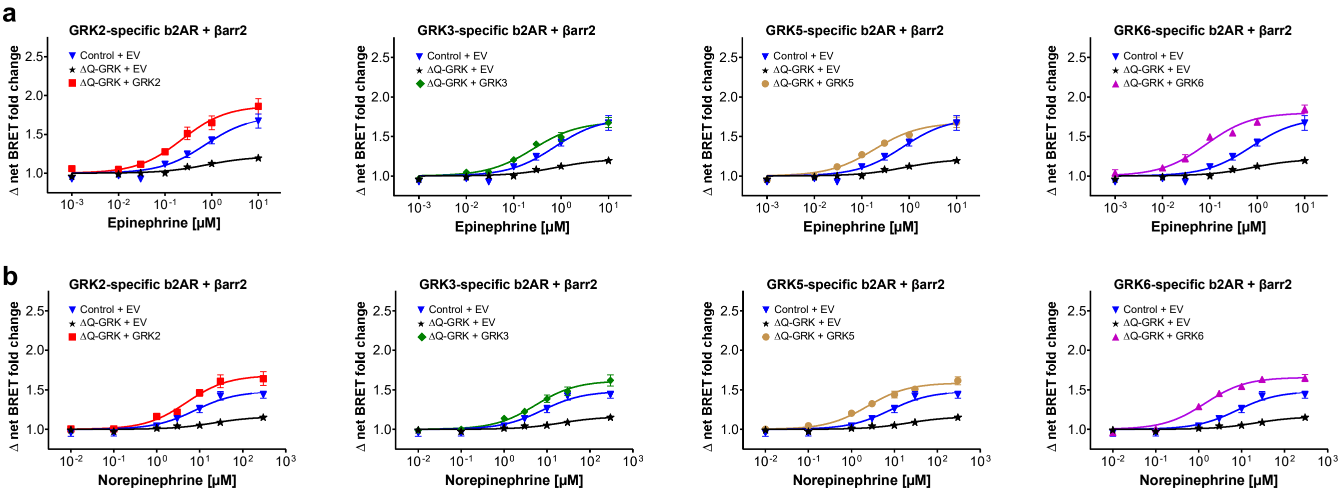

Supplementary Figure 4: GRK-specific β-arrestin 2 (βarr2) recruitment to the β2 adrenergic receptor (b2AR) induced by endogenous ligands. a, b GRK-specific βarr2 recruitment to the b2AR was measured in presence of the endogenous ligands epinephrine (**a**) or norepinephrine (**b**) (correspond to Figure 2c and d, respectively). Individual GRK isoforms were overexpressed in ΔQ-GRK as indicated. For better comparability, the data for the Control + empty vector (EV) and ΔQ-GRK + EV conditions are depicted in each panel. The data are shown as Δ net BRET fold change as mean of n = 3 independent experiments ± SEM.

Supplementary Figure 5

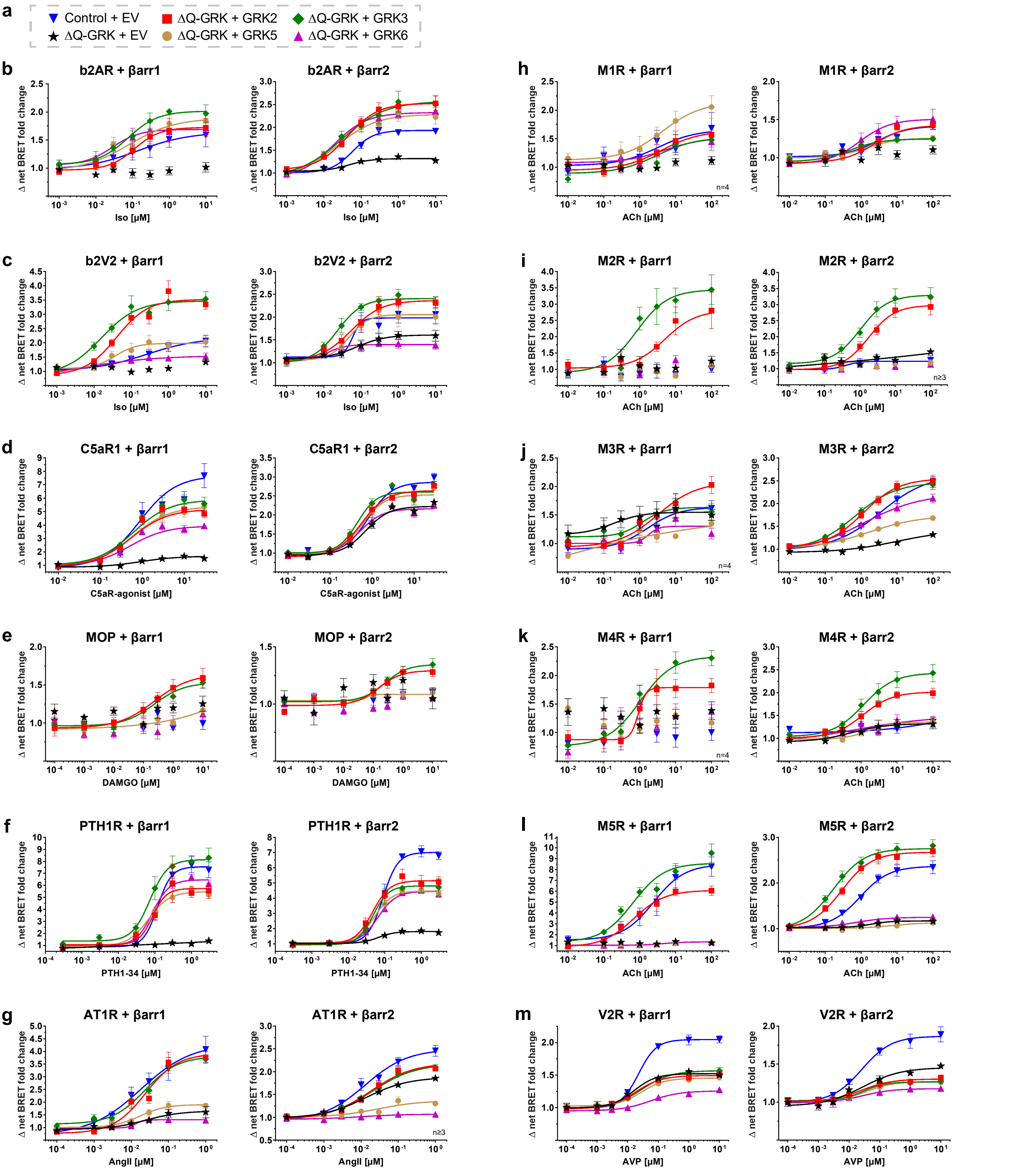

Supplementary Figure 5: Evaluation of GRK-specific β-arrestin (βarr) recruitment for twelve GPCRs. a Legend for concentration-response curves shown in (**b-m**). **b-m** The Halo-Tag-βarr fusion protein is recruited to a NanoLuciferase (NanoLuc)-tagged GPCR upon agonist activation and subsequent receptor phosphorylation. For the parathyroid hormone 1 receptor (PTH1R) and the vasopressin 2 receptor (V2R), the BRET pair was swapped. Overexpression of single GRKs in ΔQ-GRK cells allows the assessment of the impact of individual GRKs on this process. ΔQ-GRK or Control cells were transfected with the respective tagged GPCR and βarr1 or 2 fusion constructs. Additionally, either GRK2, 3, 5, 6, or empty vector (EV) were co-transfected as indicated. The dynamic BRET changes are shown as ligand concentration-response curves normalised to baseline values and vehicle control. All data points are calculated as Δ net BRET fold change as the mean of *n* = 3 independent experiments (exceptions are indicated in the respective panels) ± SEM. Results of the statistical analysis are listed in Supplementary Table 3 and plotted in Figure 3i.

Supplementary Figure 6

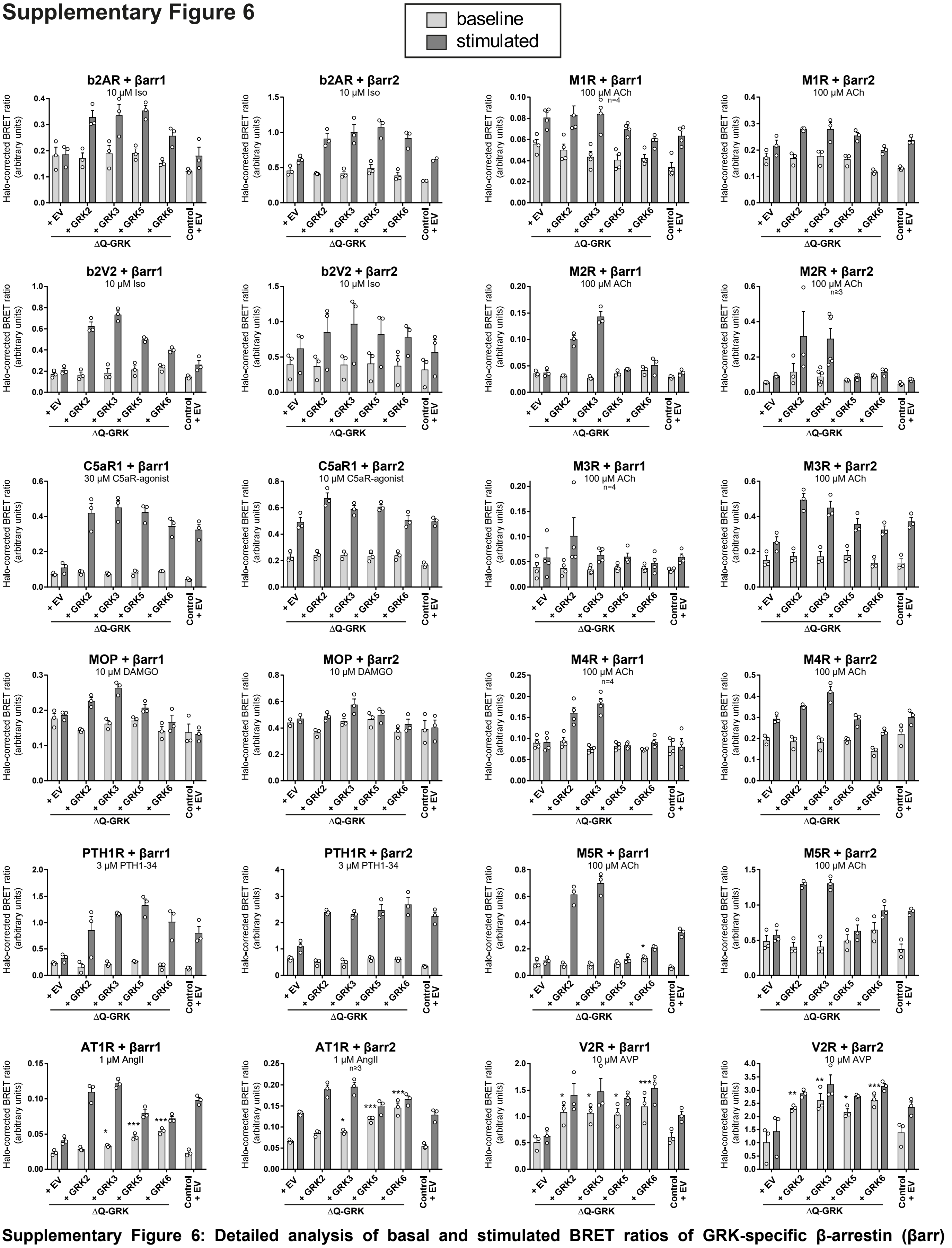

Supplementary Figure 6: Detailed analysis of basal and stimulated BRET ratios of GRK-specific β-arrestin (βarr) recruitment to twelve GPCRs. The data shown are derived from concentration-response curves in Supplementary Figure 5. ΔQ-GRK or Control cells were transfected with the NanoLuciferase (NanoLuc)-tagged GPCR and Halo-Tag-βarr1 or 2 fusion constructs. For the parathyroid hormone 1 receptor (PTH1R) and the vasopressin 2 receptor (V2R), the BRET pair was swapped. Additionally, either GRK2, 3, 5, 6, or the empty vector (EV) were co-transfected. The non-normalised, Halo-corrected BRET ratio is presented before (baseline) and after stimulation with saturating agonist concentrations (stimulated) as indicated. The bar graphs show the mean of n = 3 independent measurements (exceptions are indicated in the respective panels) ± SEM. To test whether the baseline BRET ratios were significantly elevated compared to the respective ΔQ-GRK + EV baseline, an ANOVA and one-sided Dunnett's test was performed (* $p < 0.05$; ** $p < 0.01$; *** $p < 0.001$). Baselines without indication of significance were found to be not significantly different from ΔQ-GRK + EV. All exact p values, test statistics, effect sizes, confidence intervals, and degrees of freedom are provided in the Source Data files.

Supplementary Figure 7

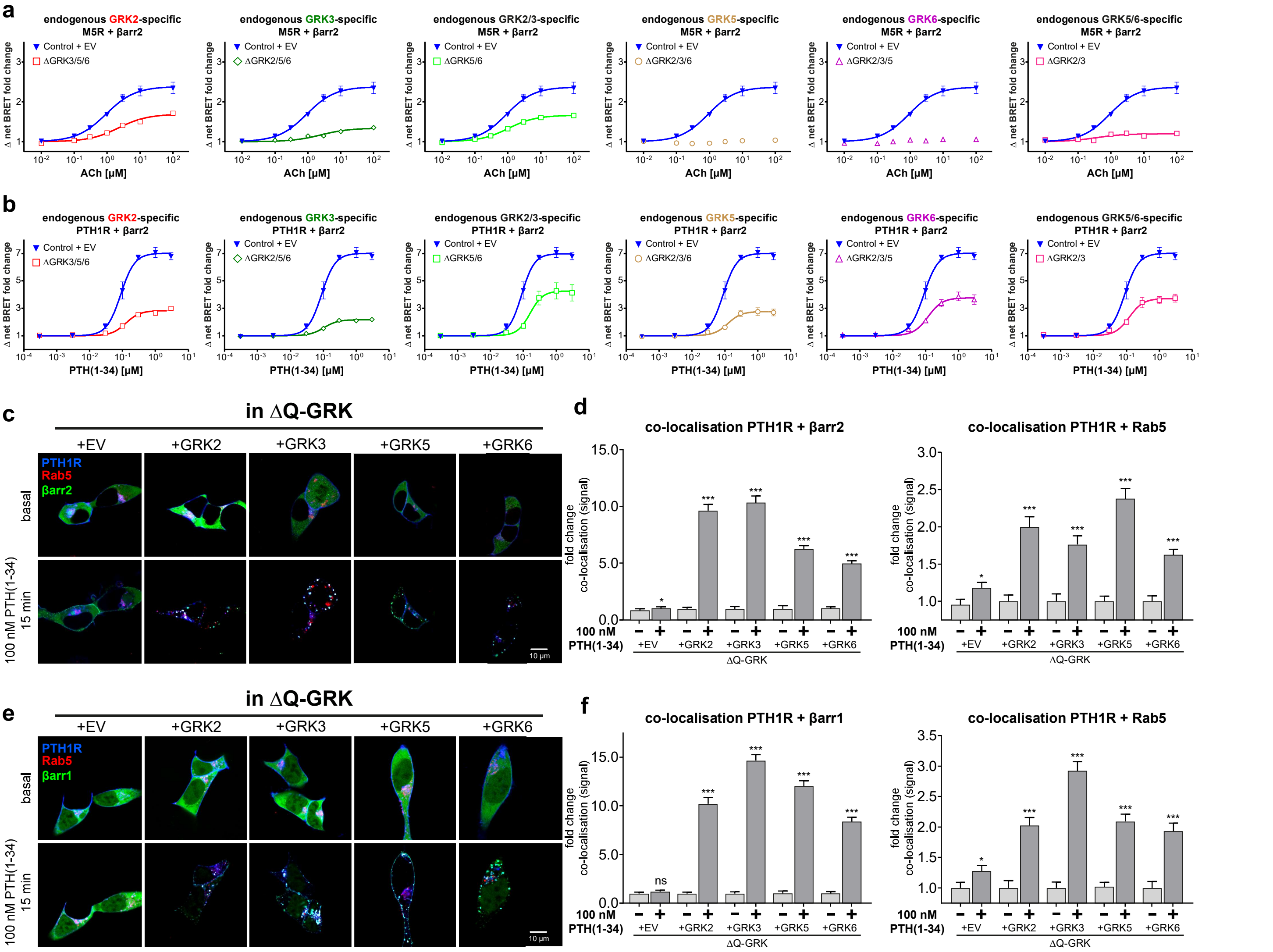

Supplementary Figure 7: GRK-specific β-arrestin2 (βarr2) recruitment to muscarinic M5 acetylcholine receptor (M5R) and parathyroid hormone 1 receptor (PTH1R) under endogenous GRK expression levels and βarr1 and 2 translocation to PTH1R with individual GRK overexpression. a, b βarr2 recruitment to the M5R (**a**) or PTH1R (**b**) in cells expressing all endogenous GRKs (Control + EV), only one remaining endogenous GRK (ΔGRK3/5/6, 2/5/6, 2/3/6, 2/3/5), or two remaining endogenous GRKs (ΔGRK5/6, 2/3) as indicated is shown as Δ net BRET fold change (mean of $n = 3$ independent experiments ± SEM). For easier comparison, Control curves are depicted in every panel. **c-f** Confocal live-cell microscopy was performed using ΔQ-GRK cells transfected with PTH1R-CFP (blue), Rab5-mCherry (red), βarr2-YFP (**c**) or βarr1-YFP (**e**) expression constructs (green) and individual untagged GRK2, 3, 5, or 6. Shown are representative images, taken before (basal) and after 15 minutes of stimulation with 100 nM parathyroid hormone (1-34) (PTH(1-34)). The normalised co-localisation of PTH1R and βarr2 or Rab5 (**d**) and βarr1 or Rab5 (**f**) was quantified using Squassh and SquasshAnalyst (number of images per respective condition; for βarr2 ΔQ-GRK + EV (52), ΔQ-GRK + GRK2 (44), ΔQ-GRK + GRK3 (41), ΔQ-GRK + GRK5 (44), ΔQ-GRK + GRK6 (51); for βarr1 ΔQ-GRK + EV (38), ΔQ-GRK + GRK2 (44), ΔQ-GRK + GRK3 (44), ΔQ-GRK + GRK5 (51), ΔQ-GRK + GRK6 (40)). Data are presented as mean fold change in co-localisation signal ± SEM. Statistical analysis was performed using a two-way mixed-model ANOVA followed by a two-sided paired t-test (* $p < 0.05$; ** $p < 0.01$; *** $p < 0.001$; **** $p < 0.0001$; ns (not significant)). All exact *p* values, test statistics, effect sizes, confidence intervals, and degrees of freedom are provided in the Source Data files.

Supplementary Figure 8

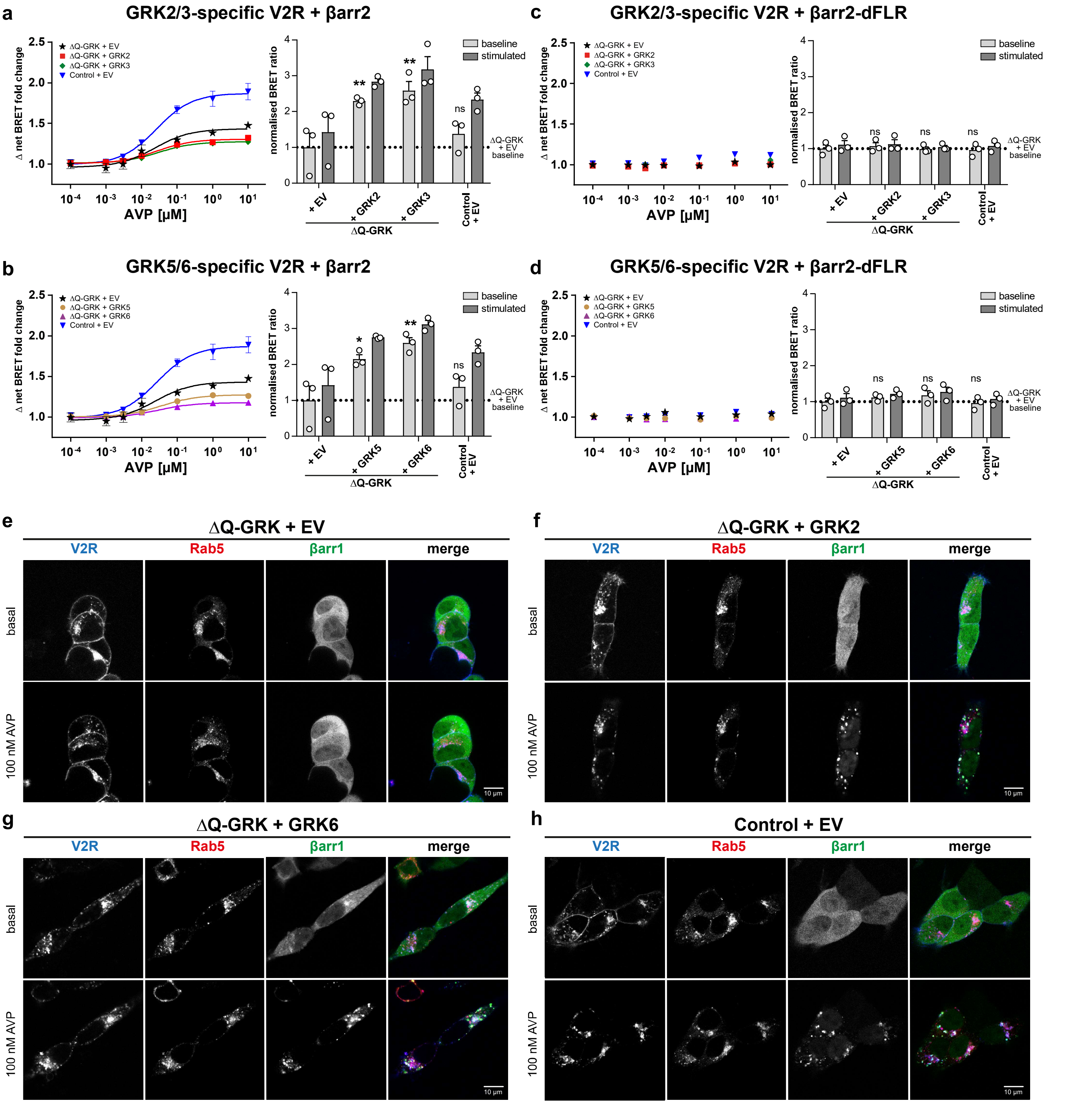

Supplementary Figure 8: GRK-specific β-arrestin 2 (βarr2) recruitment to vasopressin 2 receptor (V2R) and single channel images of βarr1 translocation to V2R. a-d ΔQ-GRK or Control cells were transfected with V2R-Halo-Tag and one of the following βarr2-NanoLuciferase (NanoLuc) fusion constructs: wild type (**a, b**) or βarr2 lacking the finger loop region (dFLR; **c, d**). Additionally, either GRK2, 3, 5, 6 or the empty vector (EV) were transfected as indicated. The dynamic BRET changes are shown as ligand concentration-response curves normalised to baseline values and vehicle control. All data points are calculated as Δ net BRET fold change as the mean of $n = 3$ independent experiments ± SEM. The same dataset is presented as bar graphs, displaying the mean BRET-values ± SEM before (baseline) and after stimulation with 10 µM [Arg8]-vasopressin (AVP; stimulated), normalised to the basal BRET ratio derived from the ΔQ-GRK + EV condition (dashed line). To test whether the baseline BRET ratios were significantly elevated compared to the respective ΔQ-GRK + EV baseline, an ANOVA and one-sided Dunnett's test was performed (* $p < 0.05$; ** $p < 0.01$; ns (not significant)). All exact p values, test statistics, effect sizes, confidence intervals, and degrees of freedom are provided in the Source Data files. **e-h** (Corresponds to Figure 4e) ΔQ-GRK or Control cells were transfected with V2R-CFP (blue), Rab5-mCherry (red), βarr1-YFP (green), and either EV, GRK2, or GRK6 as indicated. Images were taken before (basal) and after 15 minutes of 100 nM AVP stimulation (number of images per respective condition; ΔQ-GRK + EV (35), ΔQ-GRK + GRK2 (35), ΔQ-GRK + GRK6 (33), Control + EV (30)). Single channels and overlay of all three channels from the corresponding images shown in Figure 4e are depicted here.

Supplementary Figure 9

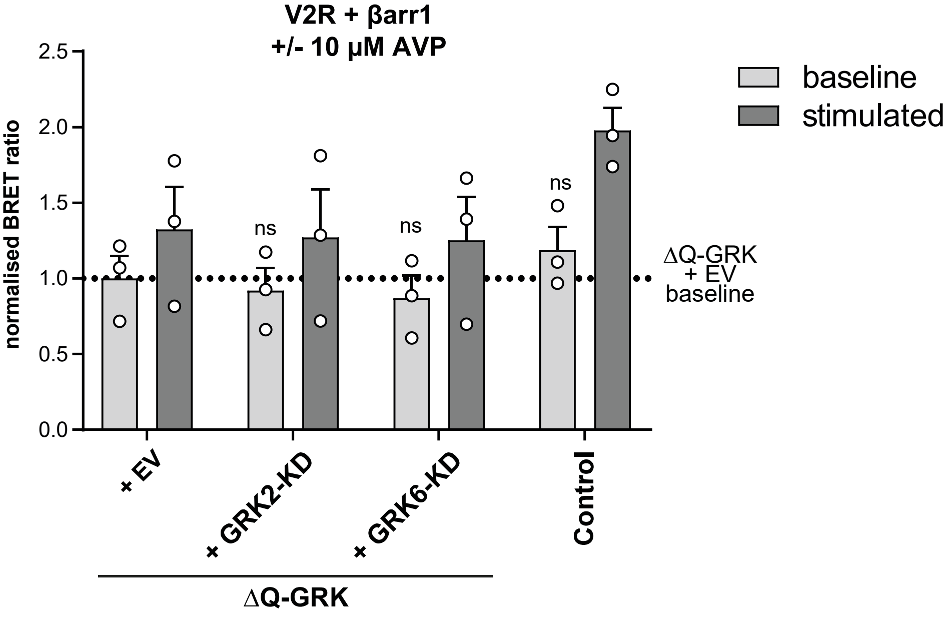

Supplementary Figure 9: β-arrestin 1 (βarr1) recruitment to vasopressin 2 receptor (V2R) with kinase-dead GRK constructs. ΔQ-GRK or Control cells were transfected with V2R-Halo-Tag and βarr1-NanoLuciferase (NanoLuc) fusion construct. Additionally, kinase-dead (KD) mutants of either GRK2 (K220R) or 6 (K215R), or the empty vector (EV) were transfected as indicated. The dataset is presented as mean of n = 3 independent experiments ± SEM in bar graphs, displaying the measured BRET-values before (baseline) and after stimulation with 10 µM [Arg8]-vasopressin (AVP; stimulated), normalised to the basal BRET ratio derived from the ΔQ-GRK + EV condition (dashed line). To test whether the baseline BRET ratios were significantly elevated compared to the respective ΔQ-GRK + EV baseline, an ANOVA and one-sided Dunnett's test was performed (ns (not significant)). All exact p values, test statistics, effect sizes, confidence intervals, and degrees of freedom are provided in the Source Data files. Recruitment data for Control + EV and ΔQ-GRK + EV were measured again in the same experiment as the GRK-KD mutants and are not identical to data presented in Figure 4a, b for these conditions.

Supplementary Figure 10

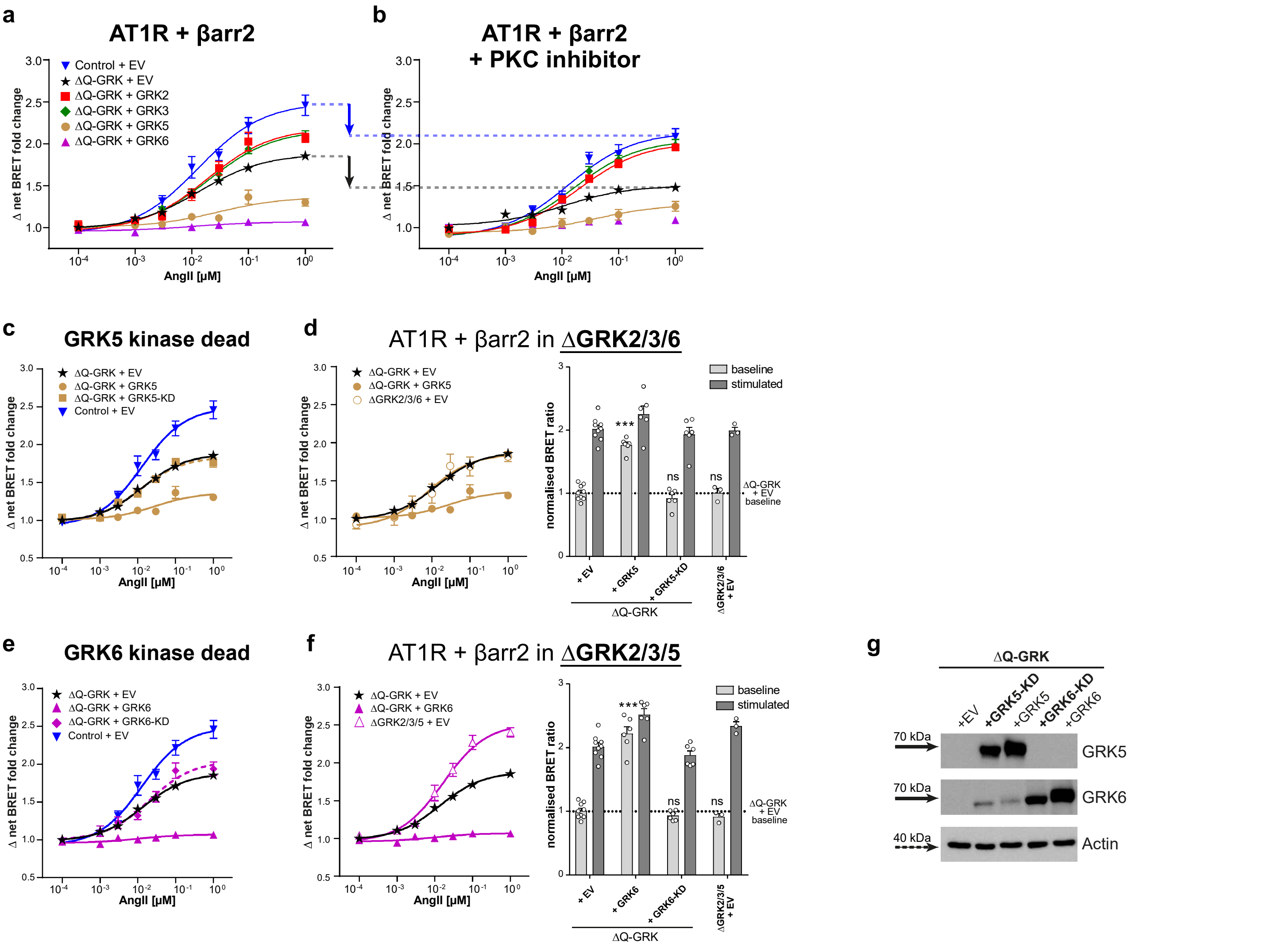

Supplementary Figure 10: β-arrestin2 (βarr2) recruitment to angiotensin 1 receptor (AT1R) with PKC inhibition, kinase dead GRK5 and 6 constructs, and under endogenous GRK5 and 6 expression. a, b ΔQ-GRK cells were transfected with AT1R-NanoLuciferase (NanoLuc), Halo-Tag-βarr2, and either one of the GRKs or the empty vector (EV) as indicated. βarr2 recruitment to the AT1R was measured in absence or presence of PKC inhibitor Gö6983 (500 nM) for one hour. Angiotensin II (AngII)-induced dynamic BRET changes are shown as concentration-response curves. To emphasise the impact of PKC on βarr2 recruitment, dynamic BRET changes of Figure 5a-f in absence (**a**) or presence of the PKC inhibitor Gö6983 (500 nM) (**b**) are shown as concentration-response curves. Downward arrows and dashed lines indicate the loss in dynamic recruitment by PKC inhibition. **c-f** Corresponding to the data shown in Figure 5e and f, ΔQ-GRK cells were transfected with AT1R-NanoLuc and Halo-Tag-βarr2. The concentration-dependent recruitment of βarr2 was measured in the presence of co-transfected GRK5 and 6 kinase-dead (KD, K215R) mutants (**c, e**) or in ΔGRK2/3/6 or ΔGRK2/3/5 (**d, f**). Recruitment data generated for Control + EV, ΔQ-GRK + EV, and ΔQ-GRK + GRK5 or 6 overexpression (Figure 5a, b, e, f) are depicted again to allow comparability. The data are also presented in respective bar graphs, displaying the mean BRET ratios ± SEM before (baseline) and after stimulation with 1 µM AngII (stimulated), normalised to the basal BRET ratio derived from ΔQ-GRK + EV (**d, f**). To test whether the baseline BRET ratios were significantly elevated compared to ΔQ-GRK + EV baseline, an ANOVA and one-sided Dunnett's test was performed (*** $p < 0.001$; ns (not significant)). All concentration-response curve data points are calculated as Δ net BRET fold change normalised to baseline values and vehicle control, represented as the mean of $n = 3$ independent experiments ± SEM. All exact p values, test statistics, effect sizes, confidence intervals, and degrees of freedom are provided in the Source Data files. **g** Representative Western blot showing the overexpression of GRK5 or 6 and GRK5- or 6-KD mutants in ΔQ-GRK.

Supplementary Figure 11

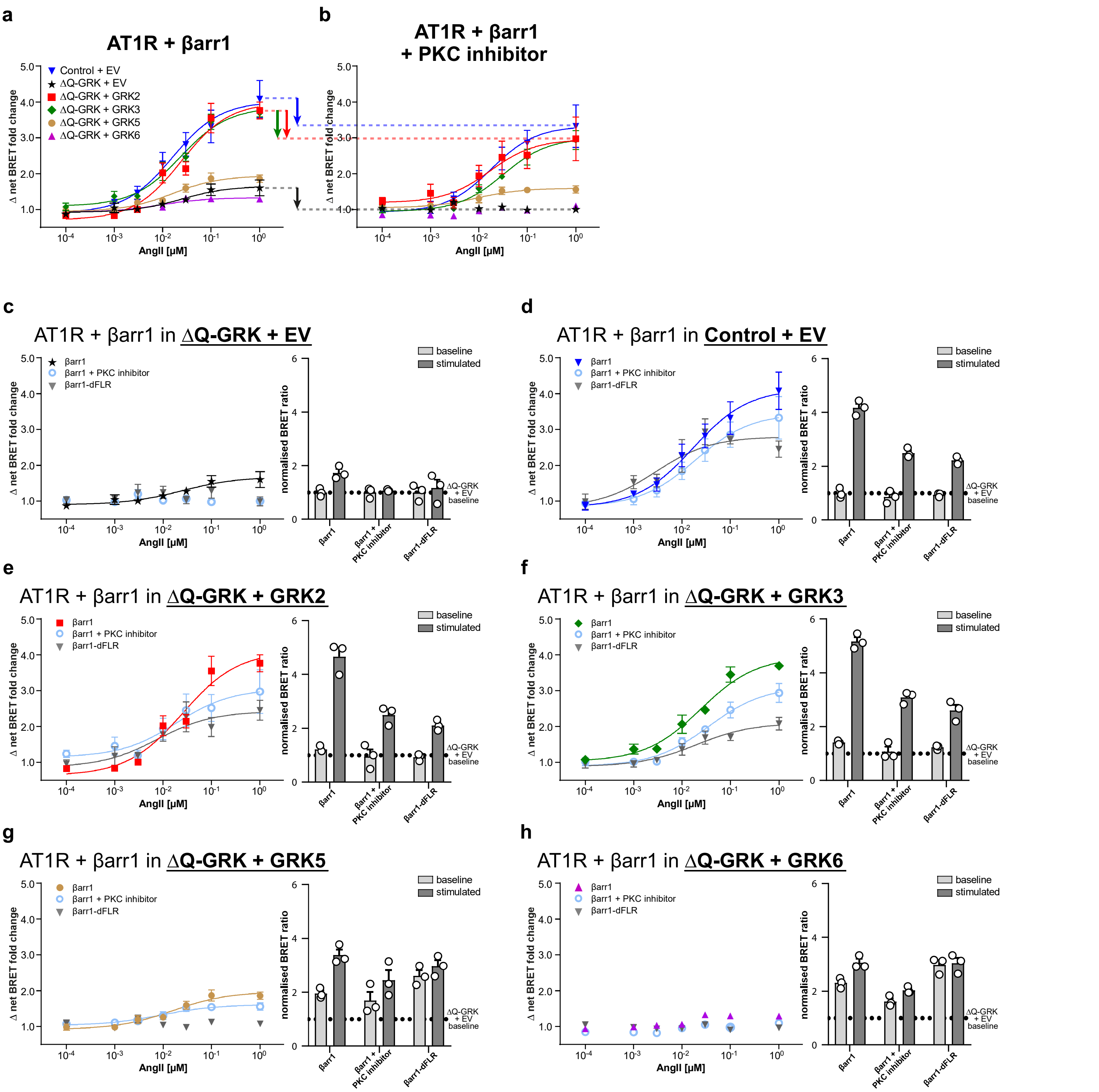

Supplementary Figure 11: GRK- and PKC-specific β-arrestin1 (βarr1) recruitment to angiotensin 1 receptor (AT1R). a, b ΔQ-GRK or Control cells were transfected with AT1R-NanoLuciferase (NanoLuc), Halo-Tag-βarr1 and either GRK2, 3, 5, 6, or the empty vector (EV) as indicated. Upon stimulation with Angiotensin II (AngII), dynamic BRET changes are shown as concentration-response curves recorded in absence (**a**) or presence of the PKC inhibitor Gö6983 (500 nM) for one hour (**b**). Downward arrows and dashed lines indicate the loss in dynamic recruitment by PKC inhibition. **c-h** To emphasise the contribution of the individual GRKs to the complex formation of βarr1 with AT1R, data shown in (**a, b**) and Supplementary Figure 5g (left panel), are now divided into smaller panels according to the individual GRK condition. Additionally, an analogous measurement using a Halo-Tag-βarr1 construct lacking the finger loop region (dFLR) was performed for each condition to test for the formation of a "hanging" complex. All data points are calculated as Δ net BRET fold change normalised to baseline values and vehicle control, represented as the mean of $n = 3$ independent experiments ± SEM. Data of these panels are also presented in respective bar graphs, displaying the mean BRET ratios ± SEM before (baseline) and after stimulation with 1 µM AngII (stimulated), normalised to the basal BRET ratio derived from the corresponding ΔQ-GRK + EV condition. The results of the statistical analysis of displayed data are listed in Supplementary Table 4. All exact p values, test statistics, effect sizes, confidence intervals, and degrees of freedom are provided in the Source Data files.

Supplementary Figure 12

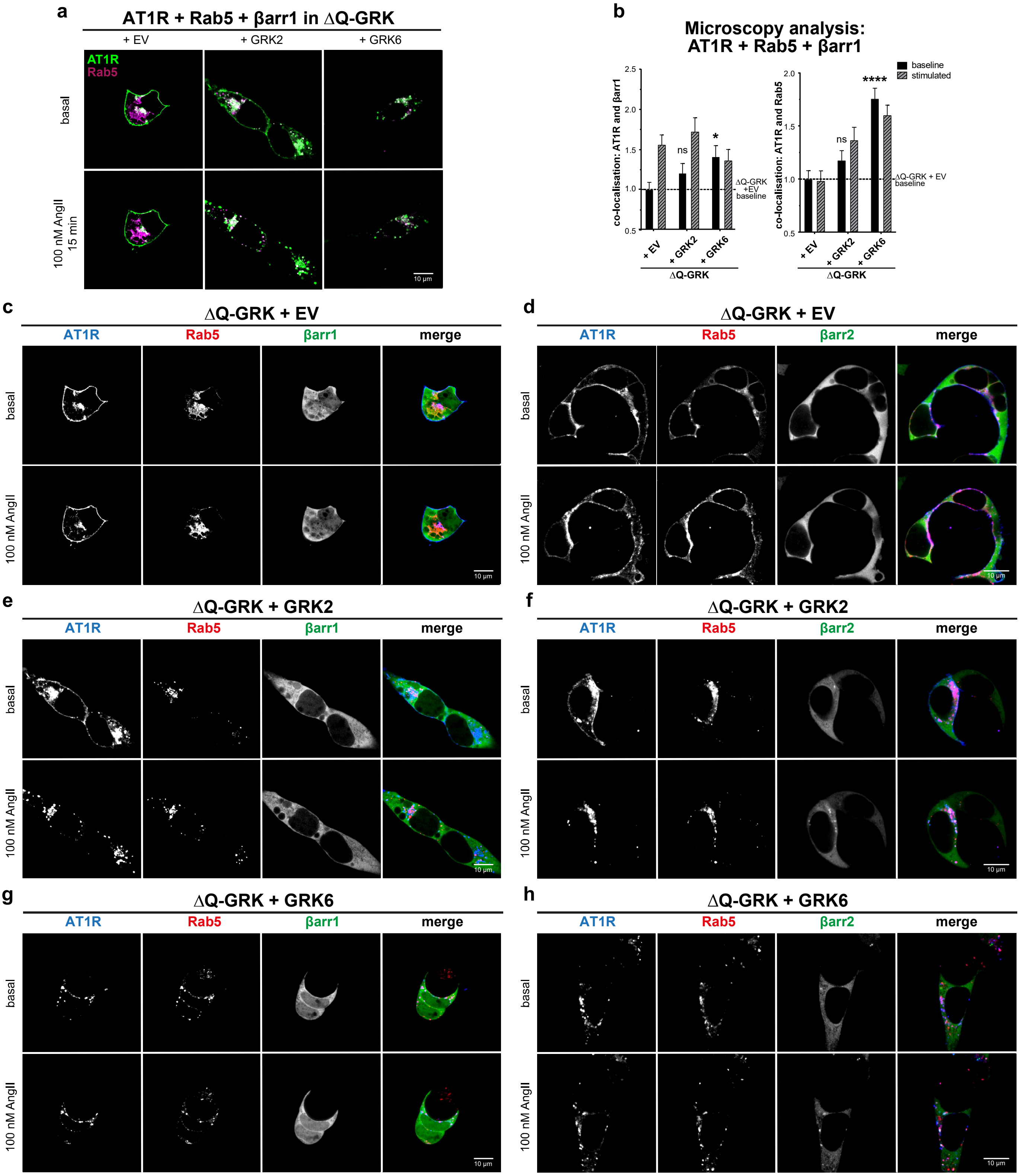

Supplementary Figure 12: β-arrestin (βarr) 1 and 2 translocation to angiotensin 1 receptor (AT1R). Corresponding to Figure 6a, b, GRK2- and 6-dependent AT1R internalisation in ΔQ-GRK is shown. **a, b** Analogous experiments were performed as depicted in Figure 6a, b, but using βarr1. In short: ΔQ-GRK cells were transfected with hsp-AT1R-CFP (green), Rab5-mCherry (magenta), βarr1-YFP, and either empty vector (EV), GRK2, or GRK6. Images were taken before (basal) and after 15 minutes of 100 nM Angiotensin II (AngII) stimulation (number of images per respective condition for βarr1; ΔQ-GRK + EV (32), ΔQ-GRK + GRK2 (31), ΔQ-GRK + GRK6 (33)). Representative images are shown in (**a**). The co-localisation of AT1R and βarr1 or Rab5 was quantified using Squassh and Squassh Analyst. Data are presented as mean fold change in co-localisation signal ± SEM, normalised to unstimulated ΔQ-GRK + EV condition (**b**, dashed line). Co-localisation under unstimulated conditions was compared using ANOVA and two-sided Dunnett's test (* $p < 0.05$; ** $p < 0.01$; *** $p < 0.001$; **** $p < 0.0001$; ns (not significant)). All exact p values, test statistics, effect sizes, confidence intervals, and degrees of freedom are provided in the Source Data files. **c-h** Depiction of single channels and overlay (merge) of hsp-AT1R-CFP (blue), Rab5-mCherry (red), and βarr1 (**c, e, g**) and 2 (**d, f, h**)-YFP (green). Additionally, cells were transfected with EV (**c, d**), GRK2 (**e, f**), or GRK6 (**g, h**). Representative images before (basal) and after 15 minutes of 100 nM AngII stimulation are shown (number of images per respective condition for βarr2; ΔQ-GRK + EV (31), ΔQ-GRK + GRK2 (31); ΔQ-GRK + GRK6 (33)).

Supplementary Figure 13

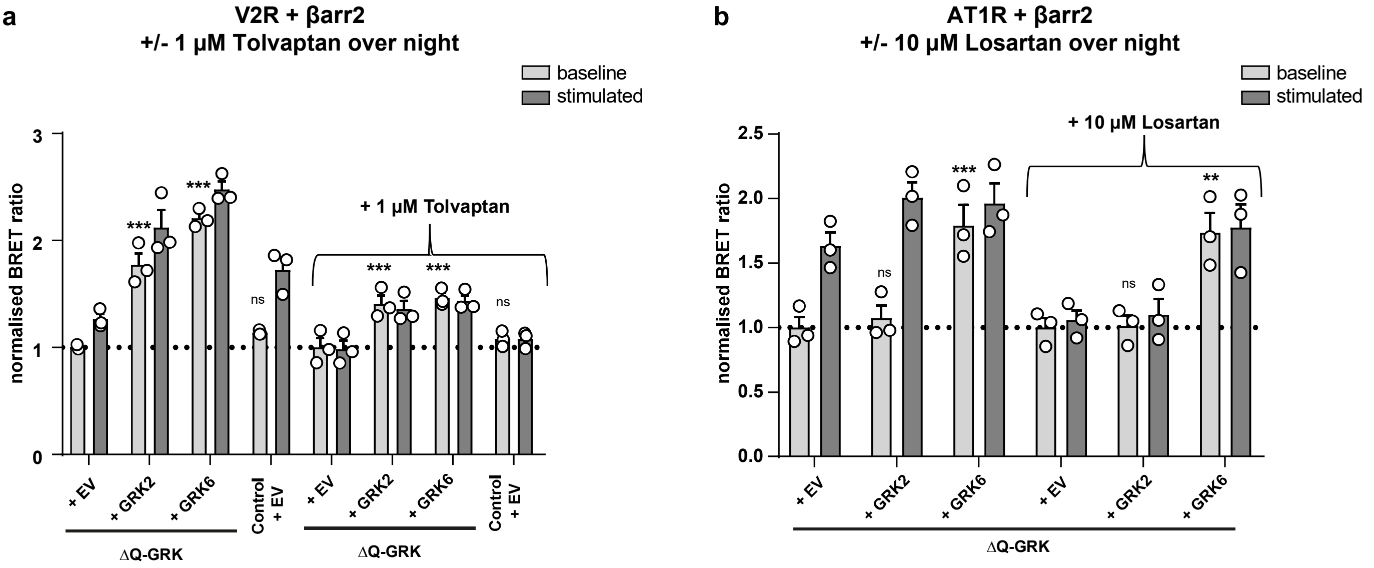

Supplementary Figure 13: Pharmacological inhibition of receptor activity does not suffice to abolish pre-coupled β-arrestin2 (βarr2) complexes with vasopressin 2 receptor (V2R) and angiotensin 1 receptor (AT1R). ΔQ-GRK or Control cells were transfected with (**a**) V2R-Halo-Tag and βarr2-NanoLuciferase (NanoLuc) fusion constructs or (**b**) AT1R-Nanoluc and β arr2-Halo-Tag plasmids. Additionally, GRK2 or 6, or the empty vector (EV) were co-transfected as indicated. 4 h after transfer into 96-well plates, the cells were pre-treated with 1 µM Tolvaptan, 10 µM Losartan, or vehicle, respectively, overnight. All washing steps prior to the measurement were conducted in presence of unchanged Tolvaptan or Losartan concentrations. The dataset is presented as mean of n = 3 independent experiments ± SEM in bar graphs, displaying the measured BRET-values before (baseline) and after stimulation with 1 µM AVP, or 1 µM AngII, respectively, normalised to the basal BRET ratio derived from the respective ΔQ-GRK + EV condition (dashed line). To test whether the baseline BRET ratios were significantly elevated compared to the respective ΔQ-GRK + EV baseline, an ANOVA and one-sided Dunnett's test was performed (** $p < 0.01$; *** $p < 0.001$; ns (not significant)). All exact p values, test statistics, effect sizes, confidence intervals, and degrees of freedom are provided in the Source Data files.

Supplementary Figure 14

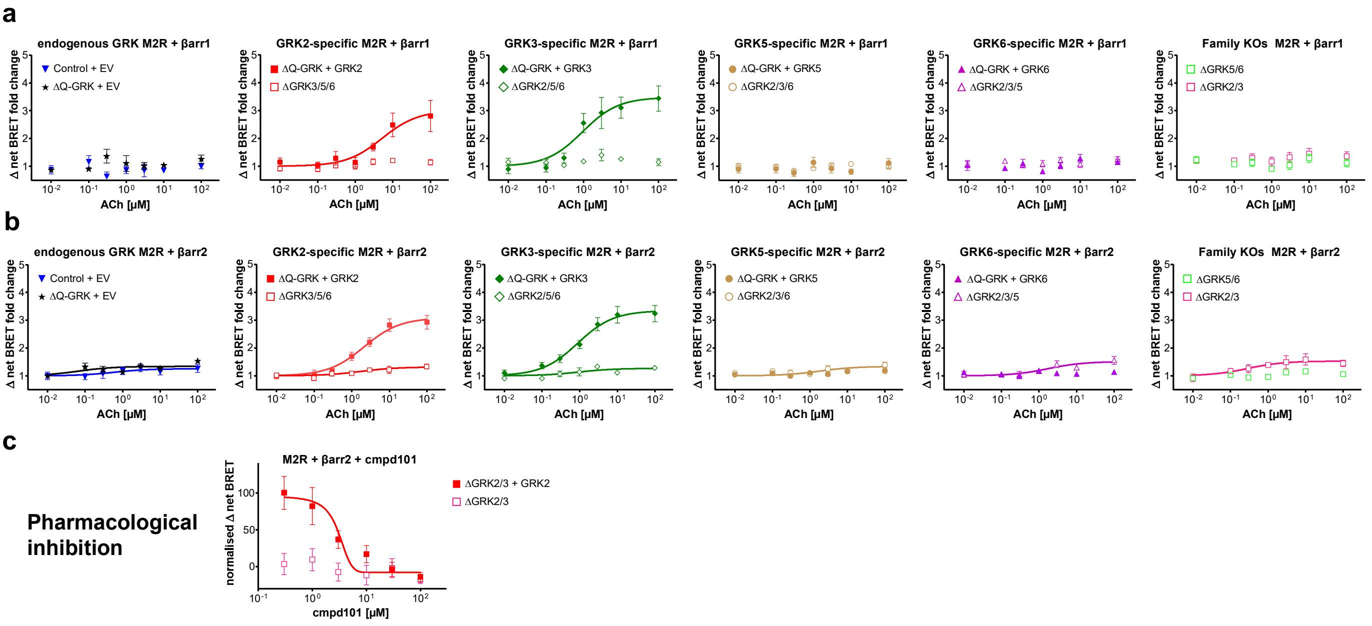

Supplementary Figure 14: GRK-specific β-arrestin (βarr)1 and 2 recruitment to muscarinic M2 acetylcholine receptor (M2R) with endogenous expression of or individually overexpressed GRKs. a, b The recruitment of βarr1 (**a**) or βarr2 (**b**) to the M2R was measured in presence of no GRK (ΔQ-GRK + empty vector (EV)), all endogenous GRKs (Control + EV), single overexpressed GRKs (ΔQ-GRK + GRK, data identical to Supplementary Figure 5i), with only one endogenous GRK left (ΔGRK3/5/6, ΔGRK2/5/6, ΔGRK2/3/6, ΔGRK2/3/5) or with a knockout of the GRK families (ΔGRK2/3 or ΔGRK5/6). Data are presented as Δ net BRET fold change (means of three independent experiments ± SEM). **c** In addition, the experiment using βarr2 was carried out in ΔGRK2/3 cells with or without overexpression of GRK2 that had been pre-incubated with different concentrations of cmpd101 for 10 minutes prior to stimulation with 100 µM of acetylcholine (ACh). Data are presented as normalised Δ net BRET as means of *n* = 3 independent experiments ± SEM.

Supplementary Figure 15

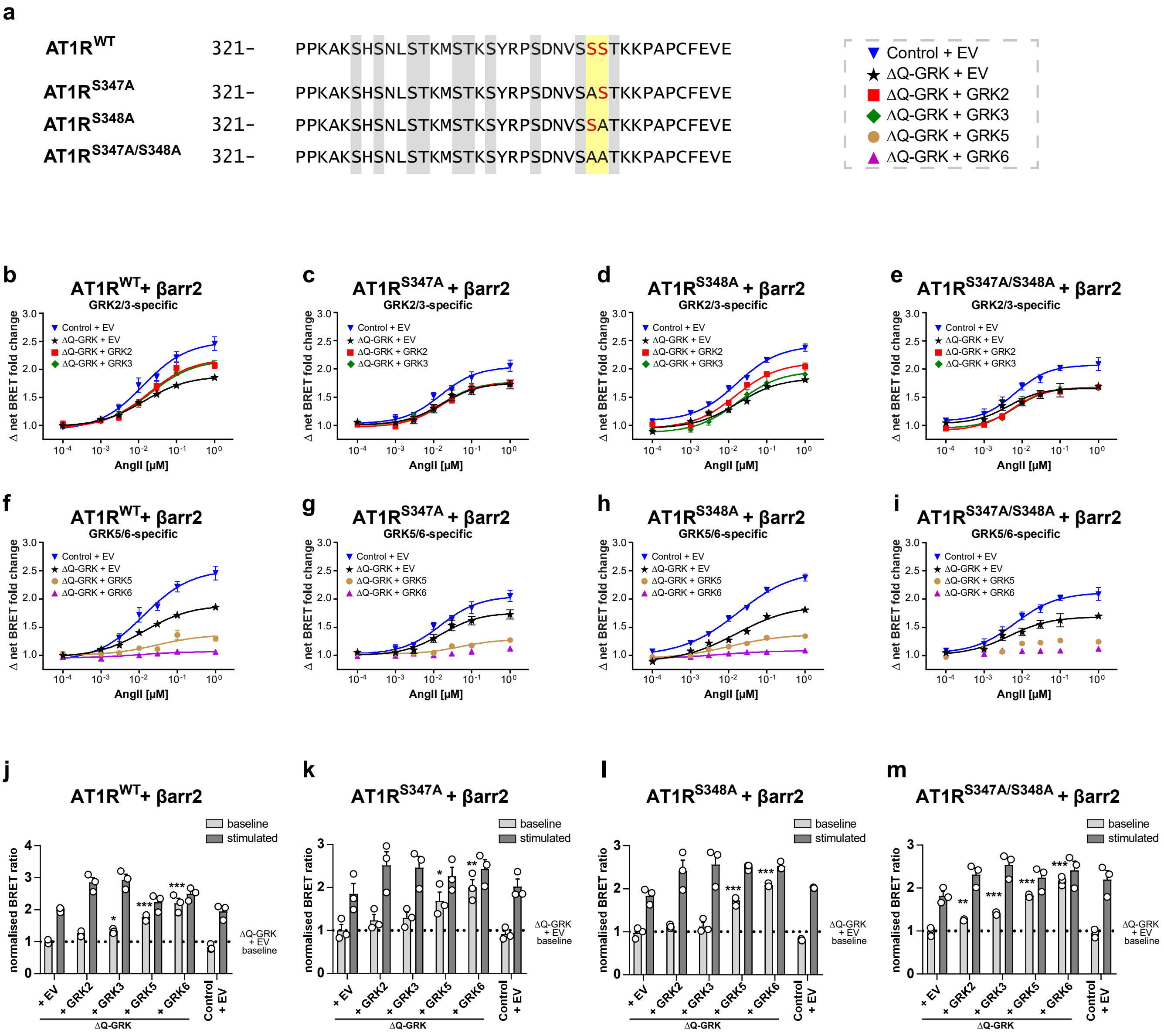

Supplementary Figure 15: GRK-specific β-arrestin 2 (βarr2) recruitment phosphorylation-deficient angiotensin 1 receptor (AT1R) mutants. a According to the proposed inhibitory phosphorylation sites by Mayer *et al.*[1], three AT1R constructs were created carrying alanine substitutions in der C-terminus using site-directed mutagenesis. Positions of proposed inhibitory sites are highlighted in yellow, other potential phosphorylation sites in grey. **b-i** ΔQ-GRK or Control cells were transfected with indicated AT1R variants carrying C-terminal Nano luciferase (NanoLuc), Halo-Tag-βarr2 and either GRK2, 3, 5, 6, or the empty vector (EV) as indicated. Upon stimulation with Angiotensin II (AngII), dynamic BRET changes are shown as concentration-response curves. All data points are calculated as Δ net BRET fold change normalised to baseline values and vehicle control, represented as the mean of n = 3 independent experiments ± SEM. Data of these panels are also presented in respective bar graphs (**j-m**), displaying the mean BRET ratios ± SEM before (baseline) and after stimulation with 1 µM AngII (stimulated), normalised to the basal BRET ratio derived from the corresponding ΔQ-GRK + EV condition. To test whether the baseline BRET ratios were significantly elevated compared to ΔQ-GRK + EV baseline, an ANOVA and one-sided Dunnett's test was performed (* $p < 0.05$; ** $p < 0.01$; *** $p < 0.001$). Data of βarr2 recruitment to AT1R wildtype (**b, f, j**) are also presented in Supplementary Figure 5g and 6. All exact p values, test statistics, effect sizes, confidence intervals, and degrees of freedom are provided in the Source Data files.

Supplementary Table 1: Results of the statistical analysis of each concentration-response curve presented. For each indicated condition the BRET ratio after ligand application in the highest concentration was compared to the recorded BRET ratio after vehicle stimulation using two-sided Student's t-test (* $p < 0.05$; ** $p < 0.01$; *** $p < 0.001$). Significant differences were considered as functional β-arrestin recruitment. All test statistics, effect sizes, confidence intervals, and degrees of freedom are provided in the Source Data files.

b2AR

βarr	condition	p value	
βarr1	ΔQ-GRK + EV	3.06E-01	
	ΔQ-GRK + GRK2	8.57E-07	***
	ΔQ-GRK + GRK3	3.10E-04	***
	ΔQ-GRK + GRK5	7.47E-06	***
	ΔQ-GRK + GRK6	1.45E-05	***
	Control + EV	8.80E-03	**
βarr2	ΔQ-GRK + EV	1.46E-05	***
	ΔQ-GRK + GRK2	3.13E-05	***
	ΔQ-GRK + GRK3	3.28E-04	***
	ΔQ-GRK + GRK5	3.20E-04	***
	ΔQ-GRK + GRK6	7.87E-07	***
	Control + EV	3.20E-09	***

b2AR + Epinephrine

βarr	condition	p value	
βarr2	ΔQ-GRK + EV	4.04E-04	***
	ΔQ-GRK + GRK2	8.51E-06	***
	ΔQ-GRK + GRK3	6.08E-07	***
	ΔQ-GRK + GRK5	6.03E-06	***
	ΔQ-GRK + GRK6	3.38E-07	***
	Control + EV	4.90E-05	***

b2AR + Norpinephrine

βarr	condition	p value	
βarr2	ΔQ-GRK + EV	6.09E-04	***
	ΔQ-GRK + GRK2	1.81E-04	***
	ΔQ-GRK + GRK3	2.42E-05	***
	ΔQ-GRK + GRK5	7.89E-09	***
	ΔQ-GRK + GRK6	7.77E-09	***
	Control + EV	9.02E-05	***

β2V2

βarr	condition	p value	
βarr1	ΔQ-GRK + EV	1.45E-01	
	ΔQ-GRK + GRK2	3.14E-07	***
	ΔQ-GRK + GRK3	2.96E-05	***
	ΔQ-GRK + GRK5	5.63E-05	***
	ΔQ-GRK + GRK6	3.78E-03	**
	Control + EV	1.66E-04	***
βarr2	ΔQ-GRK + EV	1.13E-03	**
	ΔQ-GRK + GRK2	2.96E-06	***
	ΔQ-GRK + GRK3	1.46E-06	***
	ΔQ-GRK + GRK5	1.84E-05	***
	ΔQ-GRK + GRK6	4.54E-03	**
	Control + EV	1.96E-04	***

V2R

βarr	condition	p value	
βarr1	ΔQ-GRK + EV	1.76E-04	***
	ΔQ-GRK + GRK2	3.45E-08	***
	ΔQ-GRK + GRK3	5.51E-07	***
	ΔQ-GRK + GRK5	5.40E-06	***
	ΔQ-GRK + GRK6	1.11E-04	***
	Control + EV	2.39E-11	***
βarr2	ΔQ-GRK + EV	1.14E-03	**
	ΔQ-GRK + GRK2	1.54E-07	***
	ΔQ-GRK + GRK3	3.87E-07	***
	ΔQ-GRK + GRK5	1.20E-06	***
	ΔQ-GRK + GRK6	4.90E-05	***
	Control + EV	4.38E-07	***
βarr1 - dFLR	ΔQ-GRK + EV	9.93E-01	
	ΔQ-GRK + GRK2	8.81E-01	
	ΔQ-GRK + GRK3	1.25E-02	*
	ΔQ-GRK + GRK5	1.63E-01	
	ΔQ-GRK + GRK6	8.26E-01	
	Control + EV	3.34E-03	**
βarr2 - dFLR	ΔQ-GRK + EV	8.86E-02	
	ΔQ-GRK + GRK2	3.52E-01	
	ΔQ-GRK + GRK3	9.03E-01	
	ΔQ-GRK + GRK5	7.29E-01	
	ΔQ-GRK + GRK6	7.64E-01	
	Control + EV	5.39E-02	
βarr1	ΔQ-GRK + GRK2-KD	7.45E-05	***
	ΔQ-GRK + GRK6-KD	1.39E-05	***

MOP

βarr	condition	p value	
βarr1	ΔQ-GRK + EV	4.40E-02	*
	ΔQ-GRK + GRK2	1.48E-04	***
	ΔQ-GRK + GRK3	6.73E-05	***
	ΔQ-GRK + GRK5	4.99E-02	*
	ΔQ-GRK + GRK6	3.86E-01	
	Control + EV	9.38E-01	
βarr2	ΔQ-GRK + EV	9.50E-01	
	ΔQ-GRK + GRK2	2.11E-05	***
	ΔQ-GRK + GRK3	1.17E-05	***
	ΔQ-GRK + GRK5	4.76E-02	*
	ΔQ-GRK + GRK6	1.80E-01	
	Control + EV	7.20E-02	

M1R

βarr	condition	p value	
βarr1	ΔQ-GRK + EV	2.28E-01	
	ΔQ-GRK + GRK2	1.61E-05	***
	ΔQ-GRK + GRK3	6.74E-05	***
	ΔQ-GRK + GRK5	7.13E-05	***
	ΔQ-GRK + GRK6	3.12E-02	*
	Control + EV	7.58E-03	**

M1R

βarr	condition	p value	
βarr2	ΔQ-GRK + EV	1.26E-01	
	ΔQ-GRK + GRK2	1.49E-04	***
	ΔQ-GRK + GRK3	4.19E-05	***
	ΔQ-GRK + GRK5	5.44E-05	***
	ΔQ-GRK + GRK6	1.02E-03	**
	Control + EV	5.53E-05	***

M2R

βarr	condition	p value	
βarr1	ΔQ-GRK + EV	3.06E-01	
	ΔQ-GRK + GRK2	7.08E-03	**
	ΔQ-GRK + GRK3	6.21E-07	***
	ΔQ-GRK + GRK5	6.08E-01	
	ΔQ-GRK + GRK6	3.32E-01	
	Control + EV	4.60E-01	
βarr2	ΔQ-GRK + EV	2.99E-05	***
	ΔQ-GRK + GRK2	3.24E-05	***
	ΔQ-GRK + GRK3	9.80E-08	***
	ΔQ-GRK + GRK5	1.61E-01	
	ΔQ-GRK + GRK6	3.37E-02	*
	Control + EV	1.06E-02	*
βarr1	ΔGRK3/5/6	2.53E-01	
	ΔGRK2/5/6	3.29E-01	
	ΔGRK2/3/6	7.45E-01	
	ΔGRK2/3/5	4.41E-01	
	ΔGRK5/6	5.70E-01	
	ΔGRK2/3	2.90E-01	
βarr2	ΔGRK3/5/6	3.76E-03	**
	ΔGRK2/5/6	8.87E-03	**
	ΔGRK2/3/6	6.28E-03	**
	ΔGRK2/3/5	6.00E-04	***
	ΔGRK5/6	4.02E-01	
	ΔGRK2/3	3.01E-03	**

M3R

βarr	condition	p value	
βarr1	ΔQ-GRK + EV	1.43E-02	*
	ΔQ-GRK + GRK2	1.71E-04	***
	ΔQ-GRK + GRK3	3.54E-03	**
	ΔQ-GRK + GRK5	1.42E-03	**
	ΔQ-GRK + GRK6	2.45E-02	*
	Control + EV	9.20E-04	***
βarr2	ΔQ-GRK + EV	8.03E-05	***
	ΔQ-GRK + GRK2	1.99E-07	***
	ΔQ-GRK + GRK3	1.47E-06	***
	ΔQ-GRK + GRK5	1.04E-07	***
	ΔQ-GRK + GRK6	1.29E-08	***
	Control + EV	1.34E-07	***

M4R

βarr	condition	p value	
βarr1	ΔQ-GRK + EV	7.82E-02	
	ΔQ-GRK + GRK2	1.51E-05	***
	ΔQ-GRK + GRK3	1.49E-08	***
	ΔQ-GRK + GRK5	2.66E-01	
	ΔQ-GRK + GRK6	5.22E-02	
	Control + EV	6.88E-01	
βarr2	ΔQ-GRK + EV	6.80E-03	**
	ΔQ-GRK + GRK2	1.68E-05	***
	ΔQ-GRK + GRK3	4.57E-06	***
	ΔQ-GRK + GRK5	4.44E-03	**
	ΔQ-GRK + GRK6	4.29E-03	**
	Control + EV	1.60E-02	*

M5R

βarr	condition	p value	
βarr1	ΔQ-GRK + EV	1.32E-01	
	ΔQ-GRK + GRK2	4.84E-05	***
	ΔQ-GRK + GRK3	6.51E-06	***
	ΔQ-GRK + GRK5	4.60E-02	*
	ΔQ-GRK + GRK6	7.27E-03	**
	Control + EV	1.33E-04	***
βarr2	ΔQ-GRK + EV	5.00E-03	**
	ΔQ-GRK + GRK2	2.34E-07	***
	ΔQ-GRK + GRK3	8.03E-06	***
	ΔQ-GRK + GRK5	3.88E-02	*
	ΔQ-GRK + GRK6	2.94E-03	**
	Control + EV	2.61E-04	***
βarr2	ΔGRK3/5/6	1.98E-09	***
	ΔGRK2/5/6	2.99E-05	***
	ΔGRK2/3/6	3.20E-01	
	ΔGRK2/3/5	2.29E-01	
	ΔGRK5/6	1.61E-12	***
	ΔGRK2/3	1.92E-04	***

C5aR1

βarr	condition	p value	
βarr1	ΔQ-GRK + EV	9.31E-03	**
	ΔQ-GRK + GRK2	1.27E-05	***
	ΔQ-GRK + GRK3	4.46E-05	***
	ΔQ-GRK + GRK5	1.55E-05	***
	ΔQ-GRK + GRK6	3.87E-09	***
	Control + EV	6.52E-05	***
βarr2	ΔQ-GRK + EV	6.91E-08	***
	ΔQ-GRK + GRK2	1.32E-07	***
	ΔQ-GRK + GRK3	2.11E-07	***
	ΔQ-GRK + GRK5	4.09E-07	***
	ΔQ-GRK + GRK6	1.38E-06	***
	Control + EV	5.39E-09	***

PTH1R

βarr	condition	p value	
βarr1	ΔQ-GRK + EV	8.94E-03	**
	ΔQ-GRK + GRK2	1.58E-05	***
	ΔQ-GRK + GRK3	1.08E-07	***
	ΔQ-GRK + GRK5	4.39E-07	***
	ΔQ-GRK + GRK6	2.14E-07	***
	Control + EV	1.28E-04	***
βarr2	ΔQ-GRK + EV	5.53E-08	***
	ΔQ-GRK + GRK2	1.40E-05	***
	ΔQ-GRK + GRK3	3.58E-05	***
	ΔQ-GRK + GRK5	4.45E-08	***
	ΔQ-GRK + GRK6	2.53E-07	***
	Control + EV	5.35E-09	***
βarr2	ΔQ-GRK + GRK2-YFP	2.20E-07	***
	ΔQ-GRK + GRK3-YFP	1.04E-06	***
	ΔQ-GRK + GRK5-YFP	3.90E-06	***
	ΔQ-GRK + GRK5-YFP	1.76E-07	***
βarr2	ΔGRK3/5/6	3.74E-07	***
	ΔGRK2/5/6	2.34E-08	***
	ΔGRK2/3/6	1.44E-04	***
	ΔGRK2/3/5	5.82E-05	***
	ΔGRK5/6	1.02E-03	**
	ΔGRK2/3	1.11E-03	**

AT1R

βarr	condition	p value	
βarr1	ΔQ-GRK + EV	1.21E-03	**
	ΔQ-GRK + GRK2	1.56E-05	***
	ΔQ-GRK + GRK3	1.15E-13	***
	ΔQ-GRK + GRK5	9.57E-06	***
	ΔQ-GRK + GRK6	4.48E-04	***
	Control + EV	8.71E-05	***
βarr2	ΔQ-GRK + EV	4.55E-21	***
	ΔQ-GRK + GRK2	9.64E-14	***
	ΔQ-GRK + GRK3	3.76E-08	***
	ΔQ-GRK + GRK5	7.13E-07	***
	ΔQ-GRK + GRK6	6.81E-03	**
	Control + EV	5.56E-16	***
βarr1 +PKC inhibitor	ΔQ-GRK + EV	9.40E-01	
	ΔQ-GRK + GRK2	9.89E-03	**
	ΔQ-GRK + GRK3	1.72E-05	***
	ΔQ-GRK + GRK5	1.58E-04	***
	ΔQ-GRK + GRK6	1.13E-01	
	Control + EV	1.88E-05	***
βarr2 +PKC inhibitor	ΔQ-GRK + EV	2.58E-05	***
	ΔQ-GRK + GRK2	5.15E-12	***
	ΔQ-GRK + GRK3	6.82E-08	***
	ΔQ-GRK + GRK5	2.61E-04	***
	ΔQ-GRK + GRK6	1.44E-01	
	Control + EV	1.53E-14	***
βarr1 - dFLR	ΔQ-GRK + EV	9.93E-01	
	ΔQ-GRK + GRK2	1.15E-03	**
	ΔQ-GRK + GRK3	2.77E-05	***
	ΔQ-GRK + GRK5	2.59E-01	
	ΔQ-GRK + GRK6	4.32E-01	
	Control + EV	5.41E-04	***
βarr2 - dFLR	ΔQ-GRK + EV	2.66E-01	
	ΔQ-GRK + GRK2	2.99E-05	***
	ΔQ-GRK + GRK3	1.45E-05	***
	ΔQ-GRK + GRK5	5.19E-01	
	ΔQ-GRK + GRK6	6.08E-01	
	Control + EV	1.42E-04	***
βarr2	ΔQ-GRK + GRK5-KD	9.73E-12	***
	ΔQ-GRK + GRK6-KD	2.26E-10	***
βarr2	ΔGRK2/3/6	3.52E-05	***
	ΔGRK2/3/5	1.40E-11	***

AT1R-S347A

βarr	condition	p value	
βarr2	ΔQ-GRK + EV	7.30E-03	**
	ΔQ-GRK + GRK2	2.91E-02	*
	ΔQ-GRK + GRK3	1.08E-02	*
	ΔQ-GRK + GRK5	4.99E-03	**
	ΔQ-GRK + GRK6	1.53E-01	
	Control + EV	3.90E-05	***

AT1R-S348A

βarr	condition	p value	
βarr2	ΔQ-GRK + EV	1.39E-03	**
	ΔQ-GRK + GRK2	2.88E-02	*
	ΔQ-GRK + GRK3	2.30E-02	*
	ΔQ-GRK + GRK5	9.31E-03	**
	ΔQ-GRK + GRK6	2.62E-02	*
	Control + EV	5.84E-07	***

AT1R-S347A/S348A

βarr	condition	p value	
βarr2	ΔQ-GRK + EV	8.12E-03	**
	ΔQ-GRK + GRK2	7.90E-03	**
	ΔQ-GRK + GRK3	1.54E-02	*
	ΔQ-GRK + GRK5	6.10E-02	
	ΔQ-GRK + GRK6	2.46E-01	
	Control + EV	4.10E-02	*

Supplementary Table 2: Overview of the length (number of amino acids) of intracellular loop 3 (IL3), C-terminus (C-term), pre-dominantly coupled Gα isoform[2,3], and class (according to Oakley *et al.*[4]) for each analysed GPCR (information from GPCRdb.org) with respective numbers of putative serine and threonine (S/T) phosphorylation sites, clusters (PPP, PXPP) and patterns (PXXPXXP, PXPXXP). While X represents any amino acid, P may be a serine, threonine, or a negatively charged amino acid (glutamic acid or aspartic acid). Phosphorylation sites and motifs were detected using Python 3.8.7. Custom code can be accessed via doi: 10.5281/zenodo.5764249.

GRK-specific βarr recruitment	class	Gα	GPCR	β-arrestin	IL3							C-term						
					length	P count	P density	PPP count	PXPP count	PXPXXP count	PXXPXXP count	length	P count	P density	PPP count	PXPP count	PXPXXP count	PXXPXXP count
GRK2/3	B	$G\alpha_s$	b2V2	βarr1	24	3	13%	0	0	1	0	26	11	42%	4	2	1	1
	A	$G\alpha_i$	M2R	βarr1 + βarr2	152	31	20%	8	8	6	1	10	1	10%	0	0	0	0
	A	$G\alpha_i$	M4R	βarr1 + βarr2	156	24	15%	5	4	3	4	8	1	13%	0	0	0	0
	A	$G\alpha_q$	M5R	βarr1 + βarr2	213	40	19%	14	5	4	8	21	1	5%	0	0	0	0
	A	$G\alpha_i$	MOP	βarr1 + βarr2	5	1	20%	0	0	0	0	46	11	24%	1	0	2	1
GRK2/3/5/6	A	$G\alpha_s$	b2AR	βarr1 + βarr2	24	3	13%	0	0	1	0	72	13	18%	1	1	0	0
	B	$G\alpha_i$	C5aR1	βarr1	5	2	40%	0	0	0	0	37	11	30%	2	2	1	1
	A	$G\alpha_q$	M3R	βarr2	211	51	24%	13	5	9	4	29	1	3%	0	0	0	0
	B	$G\alpha_s$	PTH1R	βarr1 + βarr2	8	1	13%	0	0	0	0	105	21	20%	3	5	2	1
not significant	B	$G\alpha_s$	b2V2	βarr2	24	3	13%	0	0	1	0	26	11	42%	4	2	1	1
	B	$G\alpha_i$	C5aR1	βarr2	5	2	40%	0	0	0	0	37	11	30%	2	2	1	1
	A	$G\alpha_q$	M1R	βarr1 + βarr2	128	22	17%	12	4	3	3	25	3	12%	0	0	0	0
	A	$G\alpha_q$	M3R	βarr1	211	51	24%	13	5	9	4	29	1	3%	0	0	0	0
pre-coupling	B	$G\alpha_q$	AT1R	βarr1 + βarr2	3	0	0%	0	0	0	0	39	12	31%	2	1	1	2
	B	$G\alpha_s$	V2R	βarr1 + βarr2	16	2	13%	0	0	0	0	26	11	42%	4	2	1	1

min max

Supplementary Table 3: Multiple comparisons of GRK-specific β-arrestin recruitment. The p values presented in this table were used to generate the heatmap in Figure 3 i. BRET fold changes at saturating ligand concentrations of at least n = 3 independent experiments were compared using ANOVA and two-sided Bonferroni's test (* $p < 0.05$; ** $p < 0.01$; *** $p < 0.001$). All test statistics, effect sizes, confidence intervals, and degrees of freedom are provided in the Source Data files.

GPCR	βarr	ΔQ-GRK vs. GRK2			ΔQ-GRK vs. GRK3			ΔQ-GRK vs. GRK5			ΔQ-GRK vs. GRK6			ΔQ-GRK vs. Control		
		non adj.	adj.		non adj.	adj.		non adj.	adj.		non adj.	adj.		non adj.	adj.	
AT1R	βarr1	0,004	0,040	*	0,006	0,050		0,687	1,000		0,618	1,000		0,001	0,008	**
AT1R	βarr2	0,155	1,000		0,117	1,000		0,000	0,000	***	0,000	0,000	***	0,002	0,015	*
b2AR	βarr1	0,024	0,220		0,005	0,042	*	0,011	0,098		0,026	0,230		0,010	0,088	
b2AR	βarr2	0,003	0,031	*	0,004	0,034	*	0,017	0,155		0,009	0,079		0,086	0,770	
b2V2	βarr1	0,000	0,002	**	0,000	0,001	***	0,089	0,801		0,619	1,000		0,078	0,704	
b2V2	βarr2	0,064	0,573		0,064	0,572		0,271	1,000		0,570	1,000		0,216	1,000	
C5aR1	βarr1	0,000	0,001	**	0,000	0,000	***	0,000	0,002	**	0,007	0,064		0,000	0,000	***
C5aR1	βarr2	0,180	1,000		0,318	1,000		0,367	1,000		0,870	1,000		0,063	0,566	
M1R	βarr1	0,079	0,713		0,149	1,000		0,001	0,013	*	0,175	1,000		0,360	1,000	
M1R	βarr2	0,073	0,658		0,399	1,000		0,368	1,000		0,030	0,273		0,081	0,731	
M2R	βarr1	0,030	0,274		0,005	0,043	*	0,827	1,000		0,882	1,000		0,700	1,000	
M2R	βarr2	0,018	0,158		0,003	0,029	*	0,553	1,000		0,492	1,000		0,649	1,000	
M3R	βarr1	0,050	0,447		0,825	1,000		0,561	1,000		0,209	1,000		0,641	1,000	
M3R	βarr2	0,000	0,004	**	0,001	0,007	**	0,167	1,000		0,008	0,068		0,001	0,005	**
M4R	βarr1	0,092	0,828		0,002	0,014	*	0,653	1,000		0,958	1,000		0,138	1,000	
M4R	βarr2	0,007	0,066		0,000	0,002	**	0,818	1,000		0,650	1,000		0,873	1,000	
M5R	βarr1	0,001	0,009	**	0,000	0,000	***	0,977	1,000		0,929	1,000		0,000	0,000	***
M5R	βarr2	0,000	0,000	***	0,000	0,000	***	0,903	1,000		0,710	1,000		0,000	0,004	**
MOP	βarr1	0,079	0,707		0,141	1,000		0,633	1,000		0,456	1,000		0,169	1,000	
MOP	βarr2	0,017	0,152		0,004	0,035	*	0,540	1,000		0,922	1,000		0,510	1,000	
PTHR	βarr1	0,009	0,082		0,000	0,002	**	0,015	0,134		0,004	0,039	*	0,001	0,008	**
PTHR	βarr2	0,001	0,007	**	0,002	0,016	*	0,005	0,046	*	0,005	0,048	*	0,000	0,000	***
V2R	βarr1	0,894	1,000		0,480	1,000		0,740	1,000		0,029	0,260		0,000	0,001	***
V2R	βarr2	0,236	1,000		0,125	1,000		0,105	0,946		0,031	0,278		0,005	0,044	*

GPCR	βarr	Control vs. GRK2			Control vs. GRK3			Control vs. GRK5			Control vs. GRK6		
		non adj.	adj.		non adj.	adj.		non adj.	adj.		non adj.	adj.	
AT1R	βarr1	0,400	1,000		0,340	1,000		0,002	0,017	*	0,000	0,003	**
AT1R	βarr2	0,326	1,000		0,406	1,000		0,000	0,000	***	0,000	0,000	***
b2AR	βarr1	0,448	1,000		0,966	1,000		0,709	1,000		0,435	1,000	
b2AR	βarr2	0,105	0,948		0,115	1,000		0,392	1,000		0,235	1,000	
b2V2	βarr1	0,006	0,053		0,002	0,022	*	0,941	1,000		0,183	1,000	
b2V2	βarr2	0,476	1,000		0,475	1,000		0,881	1,000		0,083	0,747	
C5aR1	βarr1	0,243	1,000		0,963	1,000		0,145	1,000		0,004	0,035	*
C5aR1	βarr2	0,543	1,000		0,333	1,000		0,288	1,000		0,047	0,420	
M1R	βarr1	0,442	1,000		0,652	1,000		0,020	0,179		0,717	1,000	
M1R	βarr2	0,953	1,000		0,324	1,000		0,352	1,000		0,591	1,000	
M2R	βarr1	0,015	0,132		0,002	0,021	*	0,867	1,000		0,812	1,000	
M2R	βarr2	0,007	0,062		0,001	0,010	**	0,889	1,000		0,813	1,000	
M3R	βarr1	0,120	1,000		0,806	1,000		0,301	1,000		0,092	0,832	
M3R	βarr2	0,899	1,000		0,829	1,000		0,008	0,071		0,172	1,000	
M4R	βarr1	0,004	0,035	*	0,000	0,001	***	0,338	1,000		0,151	1,000	
M4R	βarr2	0,010	0,089		0,000	0,002	**	0,943	1,000		0,768	1,000	
M5R	βarr1	0,041	0,366		0,349	1,000		0,000	0,000	***	0,000	0,000	***
M5R	βarr2	0,187	1,000		0,086	0,772		0,000	0,004	**	0,001	0,009	**
MOP	βarr1	0,005	0,049	*	0,010	0,093		0,350	1,000		0,502	1,000	
MOP	βarr2	0,058	0,525		0,014	0,122		0,962	1,000		0,573	1,000	
PTHR	βarr1	0,222	1,000		0,472	1,000		0,147	1,000		0,396	1,000	
PTHR	βarr2	0,039	0,351		0,017	0,149		0,006	0,052		0,006	0,050	*
V2R	βarr1	0,000	0,001	***	0,000	0,002	**	0,000	0,000	***	0,000	0,000	***
V2R	βarr2	0,001	0,005	**	0,000	0,002	**	0,000	0,002	**	0,000	0,001	***

Supplementary Table 4: Complete results of statistical analysis of BRET ratios shown in Figure 5 and Supplementary Figure 11 c-h. Statistical significance was determined by two-way mixed model ANOVA followed by two-sided paired t-test comparing stimulated BRET ratios to their corresponding baseline as well as two-sided Tukey's test comparing baselines or stimulated BRET ratios between each other (* $p < 0.05$; ** $p < 0.01$; *** $p < 0.001$; **** $p < 0.0001$). All test statistics, effect sizes, confidence intervals, and degrees of freedom are provided in the Source Data files.

comparing baseline and stimulated				
**condition**	**β-arrestin**	**+ βarr**	**+ βarr-dFLR**	**+ βarr + PKC inhibitor**
Control + EV	βarr1	0.00444 **	0.00829 **	0.00300 **
ΔQ-GRK + EV	βarr1	0.00469 **	0.36109	0.57302
ΔQ-GRK + GRK2	βarr1	0.00791 **	0.00185 **	0.00744 **
ΔQ-GRK + GRK3	βarr1	0.00171 **	0.01415 *	0.00712 **
ΔQ-GRK + GRK5	βarr1	0.00395 **	0.00407 **	0.01342 *
ΔQ-GRK + GRK6	βarr1	0.01012 *	0.45936	0.00920 **
Control + EV	βarr2	0.00859 **	0.09450	0.00030 ***
ΔQ-GRK + EV	βarr2	0.00033 ***	0.86111	0.00981 **
ΔQ-GRK + GRK2	βarr2	0.00469 **	0.05126	0.00141 **
ΔQ-GRK + GRK3	βarr2	0.00601 **	0.05231	0.00347 **
ΔQ-GRK + GRK5	βarr2	0.04058 *	0.01303 *	0.02003 *
ΔQ-GRK + GRK6	βarr2	0.01930 *	0.34421	0.10205

comparing baseline and baseline				
**condition**	**β-arrestin**	**βarr vs. βarr-dFLR**	**βarr vs. βarr + PKC inhibitor**	**βarr-dFLR vs. βarr + PKC inhibitor**
Control + EV	βarr1	0.93184	0.62262	0.82231
ΔQ-GRK + EV	βarr1	1.00000	1.00000	1.00000
ΔQ-GRK + GRK2	βarr1	0.45148	0.51739	0.99077
ΔQ-GRK + GRK3	βarr1	0.50462	0.14492	0.57972
ΔQ-GRK + GRK5	βarr1	0.18168	0.69820	0.06282
ΔQ-GRK + GRK6	βarr1	0.03082 *	0.02745 *	0.00092 ***
Control + EV	βarr2	0.23070	0.50580	0.78438
ΔQ-GRK + EV	βarr2	1.00000	1.00000	1.00000
ΔQ-GRK + GRK2	βarr2	0.01233 *	0.00609 **	0.76853
ΔQ-GRK + GRK3	βarr2	0.00600 **	0.00945 **	0.92324
ΔQ-GRK + GRK5	βarr2	0.00269 **	0.00002 ****	0.00000 ****
ΔQ-GRK + GRK6	βarr2	0.11392	0.05662	0.84639

comparing stimulated and stimulated				
**condition**	**β-arrestin**	**βarr vs. βarr-dFLR**	**βarr vs. βarr + PKC inhibitor**	**βarr-dFLR vs. βarr + PKC inhibitor**
Control + EV	βarr1	0.00003 ****	0.00002 ***	0.32708
ΔQ-GRK + EV	βarr1	0.17696	0.11616	0.94195
ΔQ-GRK + GRK2	βarr1	0.00051 ***	0.00014 **	0.50894
ΔQ-GRK + GRK3	βarr1	0.00006 ****	0.00004 ***	0.18578
ΔQ-GRK + GRK5	βarr1	0.56456	0.01106	0.41309
ΔQ-GRK + GRK6	βarr1	0.99380	0.00593 **	0.00694 **
Control + EV	βarr2	0.03493 *	0.12697	0.58481
ΔQ-GRK + EV	βarr2	0.10554	0.33143	0.65260
ΔQ-GRK + GRK2	βarr2	0.00629 **	0.00779 **	0.97985
ΔQ-GRK + GRK3	βarr2	0.01525 *	0.01205 *	0.98205
ΔQ-GRK + GRK5	βarr2	0.09398	0.00124 **	0.01643 *
ΔQ-GRK + GRK6	βarr2	0.00648 **	0.01708 *	0.67218

Supplementary Table 5: gRNA sequences for targeting different GRKs. Complementary forward and reverse oligos were annealed and ligated into the BsmBI-restricted lentiCRISPR v2 vector[5] (Addgene #52961). gRNA sequence is shown in capital letters, overhangs for ligation in lowercase letters. Sequences of gRNAs were validated in Sanjana *et al.*.[5]

		Oligo forward	Oligo reverse
**GRK2**	**1**	caccg CTTCGACTCATACATCATGA	aaac TCATGATGTATGAGTCGAAGc
	**2**	caccgCTCAGTGGCACTCTTCGAGA	aaac TCTCGAAGAGTGCCACTGAGc
	**3**	caccgCGCCGCCAAGATGGCGGACC	aaacGGTCCGCCATCTTGGCGGCGc
	**4**	caccgGCACACACCTGGAAGAGATC	aaacGATCTCTTCCAGGTGTGTGCc
**GRK3**	**1**	caccgTTATGATGCCTACATCATGA	aaacTCATGATGTAGGCATCATAAc
	**2**	caccgCTGATTGAAAATCTTGTCAA	aaacTTGACAAGATTTTCAATCAGc
	**3**	caccgGCTCGCCAACATGGCGGACC	aaacGGTCCGCCATGTTGGCGAGCc
	**4**	caccgTTATTTCATTTCTCTCTGCA	aaacTGCAGAGAGAAATGAAATAAc
**GRK5**	**1**	caccgCGGAAAAGCAGCCTCCCGAT	aaacATCGGGAGGCTGCTTTTCCGc
	**2**	caccgGTTCTCCCCTCAGGTACTCG	aaacCGAGTACCTGAGGGGAGAACc
	**3**	caccgAGAACCATTCCACGAATATC	aaacGATATTCGTGGAATGGTTCTc
	**4**	caccgGCCAGTGTGAAGACCTCCGA	aaacTCGGAGGTCTTCACACTGGCc
**GRK6**	**1**	caccgCGGCAAAAGGGGCCACGCTC	aaacGAGCGTGGCCCCTTTTGCCGc
	**2**	caccgCATGCCTTCCGCTTGTCATC	aaacGATGACAAGCGGAAGGCATGc
	**3**	caccgATGACAAGCGGAAGGCATGT	aaacACATGCCTTCCGCTTGTCATc
	**4**	caccgCAATACCGAGTCCTGGGCAA	aaacTTGCCCAGGACTCGGTATTGc

Supplementary References

1 Mayer, D. et al. Distinct G protein-coupled receptor phosphorylation motifs modulate arrestin affinity and activation and global conformation. Nat Commun 10, 1261, doi:10.1038/s41467-019-09204-y (2019).

2 Inoue, A. et al. Illuminating G-Protein-Coupling Selectivity of GPCRs. Cell 177, 1933-1947 e1925, doi:10.1016/j.cell.2019.04.044 (2019).

3 Sarma, J. V. & Ward, P. A. New developments in C5a receptor signaling. Cell Health Cytoskelet 4, 73-82, doi:10.2147/CHC.S27233 (2012).

4 Oakley, R. H., Laporte, S. A., Holt, J. A., Barak, L. S. & Caron, M. G. Association of beta-arrestin with G protein-coupled receptors during clathrin-mediated endocytosis dictates the profile of receptor resensitization. J Biol Chem 274, 32248-32257, doi:10.1074/jbc.274.45.32248 (1999).

5 Sanjana, N. E., Shalem, O. & Zhang, F. Improved vectors and genome-wide libraries for CRISPR screening. Nat Methods 11, 783-784, doi:10.1038/nmeth.3047 (2014).
