# Supplementary material for: GPCR kinase knockout cells reveal the impact of individual GRKs on arrestin binding and GPCR regulation
Source: Nat Commun. 2022 Jan 27;13:540. doi: 10.1038/s41467-022-28152-8 (PMC8795447; doi:10.1038/s41467-022-28152-8)
Supplement: Supplementary file 3 — Reporting Summary [file 41467_2022_28152_MOESM3_ESM.pdf]

nature portfolio

Corresponding author(s): Hoffmann, Carsten

Last updated by author(s): Dec 9, 2021

Reporting Summary

Nature Portfolio wishes to improve the reproducibility of the work that we publish. This form provides structure for consistency and transparency in reporting. For further information on Nature Portfolio policies, see our Editorial Policies and the Editorial Policy Checklist.

Statistics

For all statistical analyses, confirm that the following items are present in the figure legend, table legend, main text, or Methods section.

n/a	Confirmed	
☐	☒	The exact sample size (*n*) for each experimental group/condition, given as a discrete number and unit of measurement
☐	☒	A statement on whether measurements were taken from distinct samples or whether the same sample was measured repeatedly
☐	☒	The statistical test(s) used AND whether they are one- or two-sided<br>*Only common tests should be described solely by name; describe more complex techniques in the Methods section.*
☒	☐	A description of all covariates tested
☐	☒	A description of any assumptions or corrections, such as tests of normality and adjustment for multiple comparisons
☐	☒	A full description of the statistical parameters including central tendency (e.g. means) or other basic estimates (e.g. regression coefficient) AND variation (e.g. standard deviation) or associated estimates of uncertainty (e.g. confidence intervals)
☐	☒	For null hypothesis testing, the test statistic (e.g. *F*, *t*, *r*) with confidence intervals, effect sizes, degrees of freedom and *P* value noted<br>*Give P values as exact values whenever suitable.*
☒	☐	For Bayesian analysis, information on the choice of priors and Markov chain Monte Carlo settings
☒	☐	For hierarchical and complex designs, identification of the appropriate level for tests and full reporting of outcomes
☒	☐	Estimates of effect sizes (e.g. Cohen's *d*, Pearson's *r*), indicating how they were calculated

Our web collection on statistics for biologists contains articles on many of the points above.

Software and code

Policy information about availability of computer code

Data collection	Life microscopy data was obtained using an Leica SP8 with the Leica Application Suit X, Version 3.5.5.19976; plate reader data was obtained by using a BioTek Synergy Neo2 platereader with the Gen5 software Version 2.09; DMR data was obtained using a Corning Epic BT System using the software Epic Imager 2012 (Corning), Quantification of western blot images was done using Fujifilm multi Gauge software (V3.0).
Data analysis	For data analysis Microsoft office Excel version 2010, Graphpad Prism 7 (except for DMR data which were analysed using Graphpad Prism 8.4.3), ImageJ Version 1.52p, the Squassh and SquasshAnalyst Software as described in the Methods section and references by A. Rizk et al., Python 3.8.7, R 4.0.3 including ez R package by Lawrence et al. 2011, multcomp R package by Hothorn et al. 2008 and pheatmap R package by Kolde et al. 2013<br>https://github.com/mo-yoda/Drube_2021

For manuscripts utilizing custom algorithms or software that are central to the research but not yet described in published literature, software must be made available to editors and reviewers. We strongly encourage code deposition in a community repository (e.g. GitHub). See the Nature Portfolio guidelines for submitting code & software for further information.

Data

Policy information about availability of data

All manuscripts must include a data availability statement. This statement should provide the following information, where applicable:

- Accession codes, unique identifiers, or web links for publicly available datasets
- A description of any restrictions on data availability
- For clinical datasets or third party data, please ensure that the statement adheres to our policy

Source data are provided with this paper.

Field-specific reporting

Please select the one below that is the best fit for your research. If you are not sure, read the appropriate sections before making your selection.

- [x] Life sciences
- [ ] Behavioural & social sciences
- [ ] Ecological, evolutionary & environmental sciences

For a reference copy of the document with all sections, see nature.com/documents/nr-reporting-summary-flat.pdf

Life sciences study design

All studies must disclose on these points even when the disclosure is negative.

Sample size	No sample size calculation was performed. Based on our experience three to four repititions for transfection are sufficinet to study a protein-protein interaction and to identify if it occurs in cells or not. Our Plate-reader experiments were carried out in three (or more where indicated) independent experiments (independent transfections and readings) with each well representing more than 10Exp4 cells and each datapoint measured in triplicates. The quantifications of the western blots were carried out from membranes of freshly prepared lysates that were subjected to immunoprecipitations as indicated. Four sets of independent cell seedings and stimulations were prepared. The DMR data was obtained from three to four experiments with triplicate technical replicates each. The confocal microsopy experiments were repeated on three to four different days with independent transfections and multiple independent ligand stimulations per experimental day. Of these, at least 30 individual images were analysed. Again based on our experience this number is large enough to identify individual variations in cell transfections.
Data exclusions	BRET fold changes of arrestin recruitment (Supplementary Figure 5) were considered outliers if were higher and Q3 + 1.5xIQR (IQR = interquartile range) or lower than Q1 - 1.5xIQR. If this measurement was conducted more than three times (n > 3) these values were excluded.
Replication	Experiments were repeated as described above and in the methods part or figure legends. All experiments shown could be reproduced as described.
Randomization	This study is entirely based on in cellulo experiments. Thus, no allocation into experimental groups was needed which could be randomized.
Blinding	Investigators were not blinded during data collection or analysis. This was not possible since, transfection of the cells was conducted by the same investigators.

Reporting for specific materials, systems and methods

We require information from authors about some types of materials, experimental systems and methods used in many studies. Here, indicate whether each material, system or method listed is relevant to your study. If you are not sure if a list item applies to your research, read the appropriate section before selecting a response.

Materials & experimental systems

n/a	Involved in the study
[ ]	[x] Antibodies
[ ]	[x] Eukaryotic cell lines
[x]	[ ] Palaeontology and archaeology
[x]	[ ] Animals and other organisms
[x]	[ ] Human research participants
[x]	[ ] Clinical data
[x]	[ ] Dual use research of concern

Methods

n/a	Involved in the study
[x]	[ ] ChIP-seq
[x]	[ ] Flow cytometry
[x]	[ ] MRI-based neuroimaging

Antibodies

Antibodies used

anti HA-antibody (Cell signaling technology # 3724) (1:1,000),
anti-HA antibody (7TM000HA, 7TM Antibodies (Jena, Germany), 1:500
GRK2: Santa Cruz sc-13143 (1:500),
GRK3: Cell signaling technology #80362 (1:250),
GRK5: Santa Cruz, sc-518005 (1:250),
GRK6: Cell signaling technology #5878 (1:1,000),
phosphosite-specific MOP antibodies (7TM Antibodies (Jena, Germany)): anti-pT370 (7TM0319B), anti-pS375 (7TM0319C) anti-pT376 (7TM0319D) and anti-pT379 (7TM0319E), all 1:1,000
Actin: Sigma-Aldrich A5441 (1:2,000),
Western Blot secondary antibodies were purchased from SeraCare, goat anti rabbit Catalog No: 5220-0336 and goat anti mouse Catalog No: 5220-0341, both used 1:10,000
Vinculin: Biozol, BZL 03106 (1:1,000)

For ELISA experiments: peroxidase-conjugated anti-rabbit antibody (Cell Signaling technology #7074, 1:1,500),

Validation	https://www.cellsignal.com/products/primary-antibodies/ha-tag-c29f4-rabbit-mab/3724<br>https://de.7tmantibodies.com/7tm-immuno-grade-antibodies/epitope-tag-antibodies/ha/21/ha-tag-anti-ha-epitope-tag-antibody<br>GRK antibodies were validated using overexpression contructs as described in Reichel et al https://doi.org/10.1101/2021.10.26.465910. Further information from suppliers can be found here:<br>https://www.scbt.com/de/p/grk-2-antibody-c-9<br>https://www.cellsignal.de/products/primary-antibodies/grk3-d8g6v-rabbit-mab/80362<br>https://www.cellsignal.de/products/primary-antibodies/grk6-d1a4-rabbit-mab/5878<br>phospho-specific antibodies pS363, pT370, pT379, pS375, pT376 for MOP were extensively characterized in references Doll et al. 2012 and Just et al. 2013, as well as decribed here: https://7tmantibodies.com/<br>https://www.sigmaaldrich.com/catalog/product/sigma/a5441<br>https://www.cellsignal.com/products/secondary-antibodies/anti-rabbit-igg-hrp-linked-antibody/7074<br>https://www.seracare.com/AntiRabbit-IgG-HL-Antibody-PeroxidaseLabeled-5220-0336/<br>https://www.seracare.com/AntiMouse-IgG-HL-Antibody-Human-Serum-Adsorbed-and-PeroxidaseLabeled-5220-0341/<br>https://www.sigmaaldrich.com/catalog/product/sigma/sab4600054

Eukaryotic cell lines

Policy information about cell lines

Cell line source(s)	HEK293 cells were originally obtained from DSMZ Germany (ACC 305). The knockout derivatives were prepared as described in the manuscript.
Authentication	Cells were not further authenticated by the authors.
Mycoplasma contamination	All used cell lines were regularly checked for mycoplasma infection by using the LONZA MycoAlert mycoplasma detection kit (LT07-318) and were found to be negative.
Commonly misidentified lines (See ICLAC register)	We did not use commonly misidentified cell lines.
